# Does quantity equal quality? Source of protein influences American dog owner purchasing decisions more than the quantity of protein in the absence of marketing claims

**DOI:** 10.1093/jas/skag136

**Published:** 2026-05-08

**Authors:** Pawanpreet Singh, Sydney Banton, Michael von Massow, Zhe Peng, Guido Bosch, Christopher P F Marinangeli, Anna K Shoveller

**Affiliations:** Department of Animal Biosciences, University of Guelph, Guelph, ON, N1G2W1, Canada; Department of Animal Biosciences, University of Guelph, Guelph, ON, N1G2W1, Canada; Department of Food, Agricultural and Resource Economics, University of Guelph, Guelph, ON, N1G2W1, Canada; Department of Food, Agricultural and Resource Economics, University of Guelph, Guelph, ON, N1G2W1, Canada; Animal Nutrition Group, Wageningen University, Wageningen, 6708 PB, The Netherlands; Centre for Regulatory Research and Innovation, Protein Industries Canada, S4P1Y1, Canada; Department of Animal Biosciences, University of Guelph, Guelph, ON, N1G2W1, Canada

**Keywords:** protein quality, canine, dog owner, purchasing behavior, knowledge

## Abstract

Protein is the most expensive macronutrient worldwide, yet protein-related claims like “high protein” or “protein #1 ingredient” strongly influence dog food purchases. Little is known about how dog owners define protein quality (PQ) and how this knowledge shapes purchasing decisions. This study investigated how perceptions of dietary protein translate from personal to dog feeding habits, the level of PQ knowledge among dog owners, and whether protein source or amount has a greater influence on dog food choice. A 60-question survey was distributed to dog owners across the United States using Qualtrics (Utah, USA) (*n* = 691). Each respondent answered 12 choice experiment questions in which dog food options varied by protein amount (20%, 35%), protein source (peas, chicken, chicken meal), and price ($80, $95, $110). Descriptive statistics were analyzed in SPSS Statistics (Version 29, IBM Corp.), and a multinomial logit model in STATA (Version BE) was used for choice experiment analysis, with significance set at *P* < 0.05. Chicken had the greatest influence on purchasing choice, followed by chicken meal, and 35% protein, compared to a baseline diet of 20% protein and peas (*P *< 0.001). The majority (30%) of respondents believed PQ was parallel to quantity, while only 18% could correctly define PQ as the ability of an ingredient to meet the indispensable amino acid requirements of an individual. For respondents who correctly defined PQ, all protein sources positively influenced choice, while greater protein quantity negatively influenced choice (*P* < 0.001). Ultimately, protein source, not amount, drives purchasing behavior in the absence of protein-related claims on dog food.

## Teaser

Protein quality refers to an ingredient’s or diet’s ability to meet an individual’s indispensable amino acid requirements. However, most American dog owners believe protein quality is directly related to quantity and dietary claims such as “high protein” strongly influence dog food purchasing choices. However, in the absence of claims, protein source, rather than amount, has a greater influence on American dog owners’ decision to buy dog food. Dietary claims are meant to educate and guide consumer purchases; thus, standardized protein-related claims for pet food can be a path to increased consumer education and transparency in pet food products.

## Introduction

While protein is the most expensive macronutrient worldwide (Traverso and Schiavo 2020), there is a growing trend to increase crude protein (CP) content in commercial canine diets. The minimum CP recommendation for adult dogs is 18% on a dry matter basis (DMB; AAFCO 2023), however, some commercial diets contain protein levels exceeding 50%. The global pet food market is worth roughly $94.5 billion, and last year, pet owners spent around $65.8 billion on food and treats for their pets (American Pet Products Association 2025). Across the globe, the number of pet dogs continues to grow, with 106 million within European countries (FEDIAF 2024), 52 million in China and 90 million in the United States of America (USA; Chang 2025). Increasing pet ownership continues to drive expansion of the pet food industry, which plays a significant role in global protein sourcing (Markets and Markets 2023).

Dietary protein can come from both animal and plant sources, differing in their quality of protein. Protein quality (PQ) is a concept gaining recognition in the pet food industry, defined as the ability of an ingredient to meet an individual’s amino acid (AA) requirements based on levels of intake and digestibility (Millward et al. 2008). The latter is used as a proxy for bioavailability. In the USA, protein from animal sources accounts for approximately 60%–70% of protein intake for humans, indicating their meat consumption to be three times the global average (Poore and Nemecek 2018). Attaining sufficient protein in the human diet is generally not an issue from a nutritional perspective, given the variety of foods consumed. However, pet owners often anthropomorphize their pets and project their own beliefs about food quality onto their pets’ diets (Boya et al. 2015). There are challenges with translating this approach for pets, as in human nutrition, food-based dietary guidelines emphasize consumption of a variety of foods, often in the form of dietary patterns, to meet nutritional needs. In contrast, dogs often consume single-format diets like kibble, where the diets must be formulated to be nutritionally complete. Thus, dietary variety is not a common practice for how dogs are traditionally fed.

In both human and pet foods, consumer-facing claims like “high protein” (HP) are used to emphasize nutritional merits and facilitate purchase and consumption. It has been shown that nutrition and health claims can increase the choice of a product by 16% compared to an identical product without a claim (Kaur et al. 2017). This is an effect commonly known as “health halo,” whereby the presence of a claim can make a product appear healthier than it truly is (Chandon 2013). Dog owners are often more inclined to purchase food they perceive as healthy for their dogs than they are for themselves (Tesfom and Birch 2010) and, consequently, protein-related claims like “high protein” or “protein #1 ingredient” strongly influence dog food purchases (Banton et al. 2021; Acosta 2025). In pet food, protein-related claims are not standardized by amount or PQ. Therefore, an HP claim does not necessarily reflect PQ and vice versa, which may contribute to misconceptions about a dog’s nutritional needs and the overall quality of the diet.

Food choices carry personal, societal, and ethical implications (Dieterle 2022). One commonly used method for examining preferences is the discrete choice experiment (DCE), which presents respondents with hypothetical product options that vary across specific attributes. DCE questions are commonly used to help understand consumer preferences and willingness to pay (WTP) (Reviewed by Lizin et al. 2022). By forcing trade-offs, DCEs reveal the relative value consumers place on each attribute, even when they aren’t consciously aware of doing so (Lizin et al. 2022). While food choices can become habitual, they are also shaped by knowledge and preexisting perceptions (Gorton and Barjolle 2013; Nardi et al. 2019). In a previous DCE with 661 U.S. dog owners, respondents’ decision to buy a dog food was highly influenced by the presence of natural ingredients and the price of the product (Simonsen et al. 2014). However, the interaction between the amount of protein and the choice of product was not explored. Although it is recognized that protein claims can influence consumer choice due to the association with greater healthfulness, there is no data on how dog owners interpret the actual protein content listed in the guaranteed analysis or nutrition facts, or whether this influences their decisions to choose a product. Moreover, it remains unclear how much dog owners understand PQ and whether they equate higher protein amounts with better quality in the absence of explicit claims. This is an important gap to address to understand whether consumers consistently value higher protein content itself or if marketing claims alone create the perception of greater value.

The objectives of the current study were to determine whether perceptions of protein source and amount translate from personal to pet dietary choices and preferences, how knowledgeable dog owners are about PQ, and whether source or amount has a greater impact on dog food purchasing decisions. It is hypothesized that dog owners’ perception of protein sources and quantity in their diet would be similar to their perception of protein sources and quantity in their dog’s diet. Secondly, it was hypothesized that perceptions of PQ would be closely linked to quantity, with a general belief that more protein equates to higher quality. However, using a DCE, in the absence of explicit protein-related claims, it is hypothesized that the source of protein would have a greater influence than the amount of protein on the decision to purchase a dog food.

## Materials and methods

### Description of consumer survey

The survey titled “Consumer and Professional Perceptions on Protein Quality in Dog Food,” consisted of 60 questions and took approximately 15 min to complete. The questions were developed by the authors to assess current perspectives, attitudes, and habits of dog owners on dietary protein for themselves and their pets. All questions were in a multiple-choice, multiple-select, or a Likert scale ranking format. A brief description of survey questions can be reviewed herein. The complete survey can be found in the Supplementary Data.

Within the survey, respondents were asked three socio-demographic questions, inquiring about their state of residence, education level, and annual household income. Respondents were also asked seven questions to collect dog demographic information, such as their dog’s sex, breed size/type, where they acquired their dog, reason for acquiring a dog, and activity level. Respondents who owned more than one dog were instructed to answer all dog-related questions based on the dog with the earliest birthday in the calendar year.

There were four questions related to personal dietary habits, inquiring about dietary routine, whether they track their protein consumption, and the ranking of macronutrient importance.

Five questions within the survey explored concepts of PQ. To evaluate respondents’ understanding of PQ, two knowledge-based questions were included. One question was for respondents to select the correct definition of PQ, while another assessed if they could identify commonly added AAs in dog diets. In both questions, an option was provided for respondents to indicate that they did not know what PQ is or what AAs are, respectively. Using a 5-point Likert scale ranging from “definitely false” to “definitely true,” respondents were asked to rate the validity of the statements “The amino acid digestibility of protein sources is relevant to their nutritional quality,” and “Diet can play a role in increasing or decreasing the risk of disease.” Additionally, one question asked to rate their confidence in identifying an ingredient as a good source of protein for their dog using a 5-point Likert scale, with 5 indicating great confidence. Moreover, in two separate questions, respondents were asked to rate the PQ of a variety of ingredients—once for their own diet and once for their dog’s diet, using a 5-point Likert scale (ranging from “terrible” to “excellent”). These questions were used to assess how perceptions of PQ translate between human and canine diets.

Eleven questions were asked to understand the feeding and purchasing behavior of dog foods. Respondents were asked what format of dog food they currently feed, how they determine feeding amounts (e.g. following label recommendations), where they buy dog food, if they check the protein content on the product, and where they seek nutritional information about dog food. Two multiple-choice questions allowed respondents to select multiple options regarding how different marketing claims influence their purchasing decisions. Another question, allowing selection of multiple options, investigated the biggest challenges faced when selecting dog food. One question, allowing selection of multiple options, examined respondents’ willingness to feed their dog a diet that contained alternative protein sources.

To assess the value respondents place on protein amount in their dog’s diet, one question was used to understand why they believe protein is important in their dog’s diet, offering five selectable reasons and an option to indicate that they do not believe protein is important. One question inquired whether they believe the protein amount or source is more important in their dog’s diet.

This question is followed by the examination of how protein-related characteristics influence dog food purchasing decisions. DCE questions were developed using a full-factorial model in STATA (Version 18 BE, StataCorp LLC, College Station, Texas, USA). Respondents’ selections were used to assess the relative influence of protein amount, protein source, and price on product choice. Before the DCE questions, a cheap talk script (outlined in Table 1) was presented to orient participants to the contents of the DCE and reduce the implications of hypothetical bias (List 2001). After reading the cheap talk script, respondents randomly received 12 of 18 DCE questions. For these questions, the protein levels were chosen to reflect commonly available low-to-moderate and HP diets in the USA dog food market (20% and 35%, respectively). Protein sources represented the most common whole animal (chicken), animal meal (chicken meal), and plant-based (peas) options in the pet food market. Similarly, the price levels were chosen to reflect the market range for a 30 lbs bag of kibble at $80, $95, and $110 USD. For each DCE question, respondents chose between two dog food products that differed in the attributes mentioned above or selected the “prefer not to choose” option.

**Table 1:** Cheap talk script presented to respondents before the discrete choice experiment questions.

**The minimum recommended amount of protein for adult dogs is 18%. Regardless of your dog’s eating restrictions, please carefully read the following product options, and choose which one you would purchase for your dog.**

### Inclusion criteria and data collection

This survey was approved by the University of Guelph’s Research Ethics Board (REB 24-02-001). Each respondent gave written consent to use their data, and all data were analyzed anonymously. Before beginning the survey, respondents were asked to review the “Consent to Participate in Research” form, which detailed the study’s purpose, the researchers involved, procedures, potential risks and benefits, participation and withdrawal guidelines, their rights, confidentiality, and the inclusion/exclusion criteria. Respondents maintained anonymity as the researchers did not collect any identifying information.

The survey was distributed across the USA in English, using the online survey platform Qualtrics (Qualtrics XM, Utah, USA) to ensure unbiased responses and sufficiency of data. The USA was chosen because it has the largest dog population and the largest dog food manufacturing companies globally (Statista 2023). Participants were recruited independently of the research team via email through the Qualtrics platform to remove selection bias.

Inclusion criteria for participation in the survey were ≥18 years old, having a dog that is not on a therapeutic diet, actively participating in selecting their dog’s diet, and not working in the following professions: pet food formulation, veterinary medicine, veterinary technology, dog breeding, or pet retail. Two “attention-check” questions were added to ensure that the respondents were paying attention and not randomly answering questions. Each question instructed respondents to select a specific response, and failure to do so resulted in survey termination, with their data excluded from analyses. Qualtrics compensated participants who met the eligibility criteria and completed the entire survey with items such as cash, vouchers, gift cards, or reward points of their choice.

To confirm that the survey was working as anticipated, a preliminary soft launch was completed on June 21, 2024, with 30 respondents. After technical corrections were made to question sequencing, a second soft launch was completed with 20 respondents on June 24, 2024. The content of survey questions was not changed between soft launches, and the data collected from the soft launches were not used in the final analysis. The full launch of the survey was done on June 26, 2024, and all data were collected within 13 d of launching. The aim was to recruit 700 participants, with a quota of 50:50 male-female respondents and the age distribution to be representative of the current USA population.

### Statistical analyses

All descriptive data were analyzed using SPSS Statistics (Version 29, IBM Crop, North Castle, New York, USA). A two-sided test of equality for column proportions was conducted to investigate how different PQ knowledge groups perceive purchasing habits, the impact of marketing claims, feeding behavior, and acceptance of alternative protein sources. All pairwise comparisons were adjusted using the Bonferroni correction. A pairwise comparison was conducted in STATA (Version 18 BE, StataCorp LLC, College Station, Texas, USA) to examine whether there was a correlation between the strength and direction of ratings of ingredient quality for the respondent’s personal diet and the quality of that same ingredient for their dog’s diet. Perceptions of ingredients were sorted based on a Likert Scale of ordinal categories: terrible/poor, average, good, or excellent.

Multinomial logit models were run in STATA to analyze data from DCE questions while accounting for individual-specific heterogeneity. The dependent variable in all models was making a choice of product compared to choosing “prefer not to choose,” referred to as the alternative-specific constant (ASC). Predictor variables that were always included in each model were protein amount (20% vs 35%), protein source (peas, chicken, chicken meal), and price ($85, $95, $110). These categorical predictors were modeled using dummy variables. Specifically, one level of each variable was designated as the reference category. We used one dummy variable to capture the effect of protein amount. HP was a dummy variable that equaled one if the respondent selected the option with 35% protein and zero otherwise. Similarly, three dummy variables were used to capture the effect of protein sources. Chicken was a dummy variable that equaled one if the respondent selected the option with Chicken and zero otherwise. Chicken Meal was a dummy variable that equaled one if the respondent selected the option with Chicken Meal and zero otherwise. Peas was a dummy variable that equaled one if the respondent selected the option with Peas and zero otherwise. These attribute levels are summarized in Table 2. The coefficients of the variables, defined in Table 3, were used to interpret which characteristics had the greatest effect on choice–protein amount or protein source. When multiple interaction variables were included in the model, tests were conducted to compare the joint effects of each interaction variable. The base variable was excluded from the models to avoid estimation problems associated with perfect multicollinearity. Respondents who always selected “prefer not to choose” were removed from the analysis (lexicographic respondents).

**Table 2:** The attribute levels of the eighteen discrete choice experiment questions.

Attribute	Level 1	Level 2	Level 3
**Protein source**	Chicken	Chicken Meal	Peas
**Protein amount**	20%	35%	N/A
**Price**	$80	$95	$110

**Table 3:** Variable definitions in the multinomial logit model.

Variable	Definition	Values
**ASC**	Alternative specific constant. It accounts for the cases where a respondent does NOT choose to opt-out (i.e., “prefer not to choose”)	=1 if opt-out is not chosen; =0 if opt-out is chosen
**High protein**	High protein level (35%)	=1 if the product has 35% protein; =0 otherwise
**Chicken**	Chicken as first ingredient	=1 if the product has chicken; =0 otherwise
**Chicken meal**	Chicken Meal as first ingredient	=1 if the product has chicken meal; =0 otherwise
**Peas**	Peas as first ingredient	=1 if the product has peas; =0 otherwise
**Price**	Price level of the product	Quoted in USD; = $80, $95, $110

WTP was also estimated for each attribute. The mean WTP was calculated by dividing the coefficient of each variable by the coefficient of Price, obtained from the multinomial logit model. WTP, presented in the dollar amount, indicates the additional monetary value respondents placed on a specific dietary attribute relative to the benchmark product. Standard errors and confidence intervals for the WTP estimates were derived using the delta method. These measures allow for the statistical significance of the monetary valuations of each variable to be determined. The statistical significance of all analyses was set at *P* < 0.05.

## Results

### Demographics of respondents

Data from three respondents who selected “prefer not to choose” or “intersex” when asked “what is your sex” was removed due to the small sample size not being representative of this population. Additionally, respondents who completed the survey in less than five minutes or who selected “unsure” when answering if their dog was on a therapeutic diet were removed to ensure data quality. The 50:50 sex quota was met with 50.4% male and 49.6% female respondents. In total, 691 participants from across the USA met the inclusion criteria for this survey, and their data were used for analysis (Table 4).

**Table 4:** Demographic data, including sex, age, region of residence, education, and income of all respondents (*n* = 691).

Demographics	*n*	%
**Sex**		
** Male**	348	50.4
** Female**	343	49.6
**Age**		
** 18–24 years**	41	5.9
** 25–34 years**	165	23.9
** 35–44 years**	102	14.8
** 45–54 years**	117	16.9
** 55–64 years**	128	18.5
** 65+ years**	138	20.0
**Region^1^**		
** Northeast**	125	18.1
** Southeast**	203	29.3
** Midwest**	152	21.9
** Southwest**	95	13.6
** West**	116	16.7
**Education**		
** Less than a high school diploma**	18	2.6
** High school degree or equivalent (e.g. GED)**	298	43.1
** College degree**	152	22.0
** Bachelor’s degree (e.g. BA, BSc)**	154	22.3
** Master’s degree (e.g. MA, MSc, MEd)**	51	7.4
** Doctorate (e.g. PhD, EdD)**	9	1.3
** Professional degree (e.g. MD, DDS, DVM)**	9	1.3
**Combined household income**		
** $0–$24,999**	102	14.8
** $25,000–$49,999**	198	28.7
** $50,000–$74,999**	182	26.3
** $75,000–$99,999**	94	13.6
** $100,000–$124,999**	55	8.0
** $125,000–$149,999**	25	3.6
** $150,000 or more**	35	5.1

*n* = number of observations.

1 Region distribution is grouped as follows: Northeast (Connecticut, Delaware, Maine, Maryland, Massachusetts, New Hampshire, New Jersey, New York, Pennsylvania, Rhode Island); Southeast (Alabama, Arkansas, Florida, Georgia, Kentucky, Louisiana, Mississippi, North Carolina, South Carolina, Tennessee, Virginia, West Virginia); Midwest (Illinois, Indiana, Iowa, Kansas, Michigan, Minnesota, Missouri, Nebraska, Ohio, Wisconsin); Southwest (Arizona, New Mexico, Oklahoma, Texas); West (Alaska, California, Colorado, Hawaii, Idaho, Montana, Nevada, Oregon, Utah, Washington, Wyoming).

### Demographics of dogs

Demographic data for one dog per household, including sex, breed size/type, acquisition location, the purpose of getting the dog, as well as the total number of dogs per household, is reported in Table 5. Most respondents (41.4%) reported their dog to be moderately active (daily intentional activity such as walks), 32.9% reported their dog is very active, 15.1% reported their dog is minimally active, 6.3% described their dog as a couch potato, 3.5% described their dog as an athlete, and 0.4% were not sure how to describe their dog’s activity. When asked “how much physical activity (including walks) does your dog get in a typical day?”, 32.6% of respondents’ dogs reportedly get 30–60 min, 29.1% get 15–30 min, 17.1% get 60–90 min, 10.7% get 0–15 min, 9.2% get more than 90 min, and 0.9% were reported to get no activity.

**Table 5:** Demographic data for one dog per household, including sex, breed size/type, and acquisition location, as well as the total number of dogs per household and the purpose of acquiring a dog, for all respondents (*n* = 691).

Variable	*n*	%
**Sex**		
** Male**	416	60.2
** Female**	275	39.8
**Breed size**		
** Up to 8 lbs/3.6 kg**	49	7.1
** 8–22 lbs/3.6–10 kg**	188	27.2
** 22–55 lbs/10–25 kg**	221	32
** 55–100 lbs/25–45.4 kg**	197	28.5
** More than 100 lbs/45.4 kg**	36	5.2
**Breed type**		
** Pure-bred dog**	305	44.1
** Mixed-breed dog**	354	51.2
** Not sure/other**	32	4.6
**Where is dog from?**		
** Animal shelter/rescue**	228	33.0
** Breeder**	166	24.0
** Friends/family**	205	29.7
** Pet store**	33	4.8
** Stray/other^1^**	59	8.6
**Purpose of dog in household** ^2^		
** Security**	159	23.0
** Companionship**	577	83.5
** Working**	17	2.5
** Sport**	29	4.2
** Emotional/mental support**	348	50.4
** For children**	163	23.6
** Company for another pet**	135	19.5
** Leisure/fun**	311	45.0
** Other**	16	2.3
**Dogs per household**		
** 1**	441	63.8
** 2**	180	26.0
** 3**	49	7.1
** 4+**	21	3.0

*n* = number of observations.

1 Aggregated data for participants who selected “not sure” and “other” for the type of dog breed due to the small sample size.

2 Question allowed for the selection of multiple answers.

### Dietary patterns of respondents

Overall, 41.1% of respondents reported not following any specific dietary routine, while the remaining respondents adhered to at least one of the following dietary habits: omnivorous (34%), HP (11.6%), organic (9.4%), low sodium (8.1%), low sugar or sugar-free (6.5%), grain-free (6.4%), meat minimalist (6.2%), high fiber (5.5%), no processed foods (5.5%), low fat/low-calorie (4.3%), low or carbohydrate-free (3.2%), dairy-free (3%), diabetic (2.5%), kosher (2.2%), vegan (1.9%), pescatarian (1.6%), ketogenic (1.6%), halal (1.4%), raw (1.3%), other (1.3%), ovo-vegetarian (1.2%), gluten-free or celiac (1.2%), lacto-vegetarian (1%), renal or kidney-focused (1%), ovo-lacto vegetarian (0.9%), and low fiber (0.7%). Among the 19% of respondents who follow a vegetarian, vegan, or meat-minimalist diet, 34% cite health and nutrition as their primary reason for doing so, 21% choose it for animal welfare reasons, 17.3% for environmental considerations, 13.6% for taste preference, 5.2% due to dietary restrictions, and 8.9% for reasons not listed.

From a list of different methods of tracking dietary protein intake, nearly half of the respondents (49.1%) reported not monitoring their protein intake in any way. However, among those who did monitor protein intake, 20.3% reported doing so by incorporating a protein source in at least one meal daily, and 17.5% reported that they have at least one source of protein in every meal. Meanwhile, 9.1% of respondents reported tracking their protein intake in grams per day, and 4% of respondents reported that they are unaware of which foods contain protein.

Respondents were asked to allocate points to a list of dietary macro- and micronutrients based on their importance in their diet, with greater points representing greater importance. Protein received the highest average allocation (32.16 ± 16.08), followed by vitamins (22.96 ± 14.65), carbohydrates (17.19 ± 11.68), minerals (14.56 ± 9.86), and then fats (13.13 ± 9.39).

### Understanding of PQ

When respondents were asked to select a definition that they believed best described “protein quality,” the majority (30.8%) selected that “protein quality is the total amount of protein in an ingredient, such that a higher quantity of protein means better quality” (PD quantity). Meanwhile, 26.5% selected “I am not sure” (PD unsure), and 18% correctly identified PQ as “protein quality is the ability of an ingredient to meet the AA requirements of an individual” (PD correct). A similar proportion (17.6%) believed PQ refers to “the total number of AAs present in an ingredient, where a higher total indicates better quality” (PD AA). The smallest portion (7.1%) selected “protein quality is the taste and palatability of a protein-rich food, where better-tasting foods have higher protein quality” (PD taste). These protein knowledge groups will hereafter be referred to by their respective abbreviations, with PD referring to the PQ definition.

When respondents were shown a list of five dog foods containing the same first five ingredients (chicken, rice, lentils, blueberries, and fish oil) and asked to identify the diet that included the AA supplements, the majority of respondents (36.7%) reported that they were not sure what an AA was. Added lysine and methionine were correctly identified by 22.3% of participants. The option with “curcumin, thiamine mononitrate, niacin” was selected by 14.7%, 13.5% selected the option with “glucosamine, choline chloride, biotin,” and 12.8% selected an option with “thiamine mononitrate, biotin, D-calcium.” Of the participants who selected PD correct, 29.6% and 34.4% of those who selected PD AA also correctly identified the AA-supplemented option, which was greater than those in the PD unsure group (*P* < 0.001) but comparable to those in the PD quantity (22.0%) and PD taste (14.3%) groups. In total, only 5.3% of respondents answered both knowledge questions correctly, meaning that they could correctly define PQ and identify the diet with an AA supplemented, while 29.7% answered one correctly, and 65% answered neither question correctly. Respondent’s level of education did not have a significant effect on the ability to define PQ or identify AAs (*P* > 0.05) and thus was removed from the models.

For the statement “The amino acid digestibility of protein sources is relevant to their nutritional quality,” 72.7% viewed it as true, 24.4% were neutral, and 2.5% viewed it as false. Lastly, for the statement “Diet can play a role in increasing or decreasing the risk of disease,” 88.8% of respondents viewed it as true, 9.1% were neutral, and 1.7% viewed it as false.

Moreover, when asked to rate the statement “I feel like I can decipher between good and poor sources of protein for my dog” based on how well it described them, using a Likert scale ranging from “does not describe me” to “describes me extremely well”; 15.0% of respondents reported that the statement “describes me extremely well,” while 28.4% reported it described them “very well,” 31.6% “moderately well,” 16.3% “slightly well,” and 8.4% reported it does not describe them at all.

### Dog owner’s perception of PQ of protein foods in their diet

**Figure 1:**
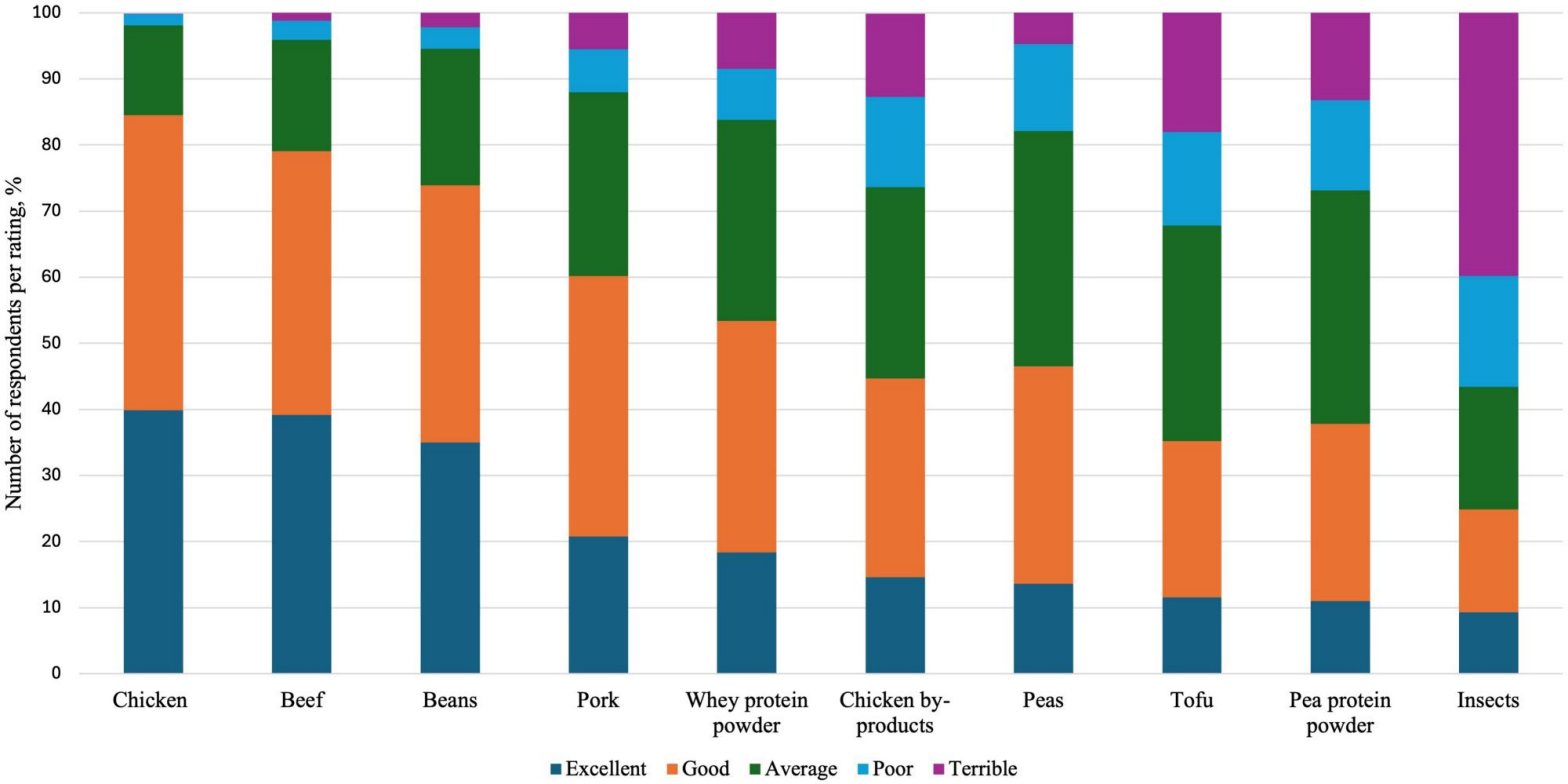
Respondents’ perceptions of protein quality for various ingredients in the human diet, presented as the percentage of total ratings for each ingredient, ordered from highest to lowest proportion of “excellent” ratings.

Dog owners were asked to rate the PQ of a variety of animal and plant-based protein ingredients. The quality of chicken as a protein source was rated as excellent by 40.1%, good by 44.5%, average by 13.5%, poor by 1.7%, and terrible by 0.1% of respondents (Figure 1). Chicken by-products were less appealing since they were rated excellent, good, average, poor, and terrible by 14.7%, 30.1%, 29.0%, 13.7%, 12.5% of respondents, respectively. Beef was perceived to have a similar PQ to chicken (excellent, 39.3%; good, 39.8%; average, 16.7%; poor, 3.0%; and terrible, 1.2%). Pork was considered to have a lower PQ than beef (excellent, 21.0%; good, 39.3%; average, 27.7%; poor, 6.5%; terrible, 5.5%). Insects had the poorest perception of PQ, rated excellent by 9.5%, good by 15.6%, average by 18.6%, poor by 16.7%, and terrible by 39.6% of respondents.

For plant-based ingredients, beans (such as chickpeas, black beans, and others) received the highest portion of excellent ratings at 35.2%, followed by 38.9% good, 20.6% average, 3.2% poor, and 2.2% terrible. In the case of peas, 13.8% of respondents rated them excellent, 32.9% good, 35.4% average, 13.1% poor, and 4.8% terrible. The ratings of tofu were similar to peas, as it received excellent ratings from 11.7% of respondents, good from 23.5%, average from 32.4%, poor from 14.3%, and terrible from 18.2%. Both whey and pea protein powders received the most ratings in the good and average categories. Whey protein powder was seen as excellent by 18.4%, good by 35.2%, average by 30.3%, poor by 7.6%, and terrible by 8.5%. Pea protein powder, perceived as lower quality than whey protein powder, was rated excellent by 11.1%, good by 26.7%, average by 35.4%, poor by 13.7%, and terrible by 13.1% of respondents.

### Dog owner’s perception of PQ of ingredients in their dog’s diet

**Figure 2:**
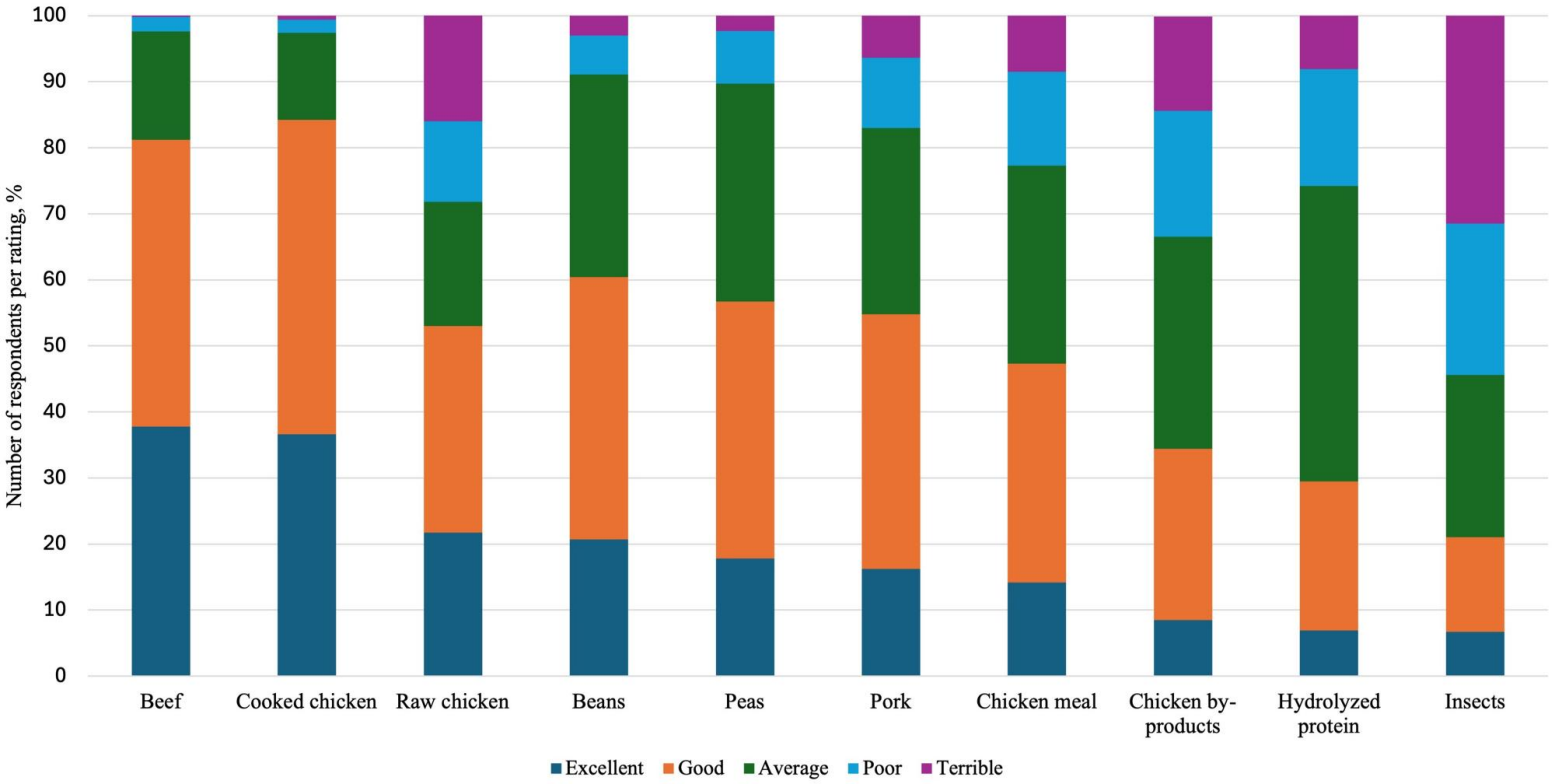
Respondents’ perceptions of protein quality for various ingredients in the canine diet, presented as the percentage of total ratings for each ingredient, ordered from highest to lowest proportion of “excellent” ratings.

When evaluating the PQ of the ingredients for dogs, cooked chicken received high ratings as it was rated excellent by 36.5%, good by 47.4%, average by 13.1%, poor by 2.0%, and terrible by 0.6% (Figure 2). Beef also had a high number of excellent ratings at 37.6%, 43.2% good, 16.3% average, 2.2% poor, and 0.3% terrible. Raw chicken was rated as excellent by 21.6%, good by 31.1%, average by 18.7%, poor by 12.1%, and terrible by 16.0%. Pork was less favored than beef as it was rated excellent by 16.1%, good by 38.5%, average by 28.1%, poor by 10.5%, and terrible by 6.3%. For chicken meal, ratings were 14.1% excellent, 33.0% good, 29.8% average, 14.1% poor, and 8.5% terrible. Chicken by-products were the least favored animal product as they were rated excellent by 8.5%, good by 25.8%, average by 32.0%, poor by 19.0%, and terrible by 14.3% of respondents. Beans (such as chickpeas, black beans, and others) were rated excellent by 20.6%, good by 39.5%, average by 30.5%, poor by 5.9%, and terrible by 3.0%. Peas received 17.7% excellent, 38.8% good, 32.9% average, 7.9% poor, and 2.3% terrible ratings. The last two ingredients, insect and hydrolyzed protein, received the lowest ratings among all ingredients. Insect protein received the lowest excellence ratings at 6.6%, with 14.3% rating it good, 24.5% average, 22.8% poor, and 31.4% terrible. Hydrolyzed protein was rated as excellent by 6.9% of respondents, good by 22.5%, average by 44.5%, poor by 17.6%, and 8.1% as terrible.

### Correlation between perceptions of PQ of protein sources in the owner’s and their dog’s diet

Perceptions of PQ for human and dog diets were positively and significantly correlated. Respondents were likely to rate an ingredient similarly for their own diet and their dog’s diet. In these analyses, chicken was compared to cooked chicken, hydrolyzed protein was compared to whey protein and pea protein separately, while all other ingredients were the same between the comparison of human and dog diets. This correlation in perception can be summarized as follows: chicken compared to cooked chicken (*r* = 0.3085, *P* < 0.0001), chicken by-product (*r* = 0.3056, *P* < 0.0001), beef (*r* = 0.5490, *P* < 0.0001), pork (*r* = 0.5375, *P* < 0.0001), beans (*r* = 0.4347, *P* < 0.0001), peas (*r* = 0.3862, *P* < 0.0001), hydrolyzed protein compared to whey (*r* = 0.2534, *P* < 0.0001) and pea protein (*r* = 0.2459, *P* < 0.0001), and insects (*r* = 0.6452; *P* < 0.0001).

### Dog feeding habits of dog owners

Respondents could report more than one feeding format for their dog. Kibble was the most frequently reported format of food (84.4%), followed by canned food (38.8%), home-cooked meals (23.0%), raw diets (13.5%), gently cooked meals (10.6%), and freeze-dried food (8.4%). Meanwhile, 4.5% of respondents reported feeding their dog a food format other than what is listed above. Additionally, a greater portion of people in the PD quantity (20.1%) and PD AA (16.4%) groups reported that they currently feed their dog a raw diet compared to the 4.3% of the PD unsure group (*P* < 0.001), while all were similar to PD taste (14.3%) and PD correct (12.0%). Moreover, a greater number of respondents in the PD taste group (20.4%) reported that they feed their dog a gently cooked diet compared to the PD unsure group (5.4%; *P* = 0.02), but both groups had similar proportions to PD quantity (13.1%), PD correct (10.4%), and PD AA (9.8%).

Next, respondents were asked to rate statements based on how well they described their dog food-feeding habits based on a Likert scale ranging from “does not describe me” to “describes me extremely well.” Firstly, 26.1% of respondents reported the statement “I follow the feeding guidelines on the label” described their feeding habits “moderately well,” followed by 24.1% reported it describes them “very well,” 20.9% reported it “does not describe [them],” 17.3% reported it described them “slightly well” and 11.2% reporting it describes them “extremely well.” The statement “I feed my dog an amount of food based on my veterinarian’s recommendation” reportedly described 25.6% of respondents “very well,” 24.8% “moderately well,” 22.8% not at all, 14.6% “slightly well” and 11.8% “extremely well.”

The majority of respondents reported that a “high protein” claim (52.2%) best described what they were currently feeding their dog. Other relevant claims included “grain-free” (22.1%), “no added supplements” (20.7%), “limited ingredient” (19.8%), “grain-inclusive” (9.3%), “plant-based diet” (5.5%), and “hydrolyzed protein” (4.6%). A small portion of respondents chose the “other” category (5.2%). Meanwhile, 23.6% of respondents reported that they do not look for claims on dog food. Among respondents who reported that they follow a HP diet themselves, 80% also fed their dog a HP diet (*P* < 0.001). Interestingly, nearly half (48.4%) of respondents who reported they do not follow an HP diet still stated feeding their dog a HP diet (*P* < 0.001). Additionally, a greater proportion of respondents who do not follow a specific dietary habit (35%) reported not looking for claims on their dog’s food, compared to those who reportedly follow a dietary habit (15.4%; *P* < 0.001).

### Dog food purchasing habits

The most frequently reported sources of getting information about dog food were online sources (57.4%), followed by veterinarians (51.6%), friends and family (35.4%), and pet stores (28.8%). Other sources included company websites (14.8%), TV (10.5%), breeders (9.5%), animal shelters or rescues (8.9%), and sources other than the ones listed (5.8%).

The most commonly reported dog food purchasing habit was to purchase the same brand and flavor of dog food every time (40.2%). This was followed by those who buy the same brand but rotate the flavors for their dog (33.1%), rotate their dog’s food often with different brands and flavors (19.7%), and respondents who reported they buy and rotate their dog’s food based on the lowest cost (3.6%). A smaller portion of respondents (2.5%) report that they prepare their dog’s meals at home using different ingredients, and 0.9% report that their purchasing habits are different from the above options.

**Figure 3:**
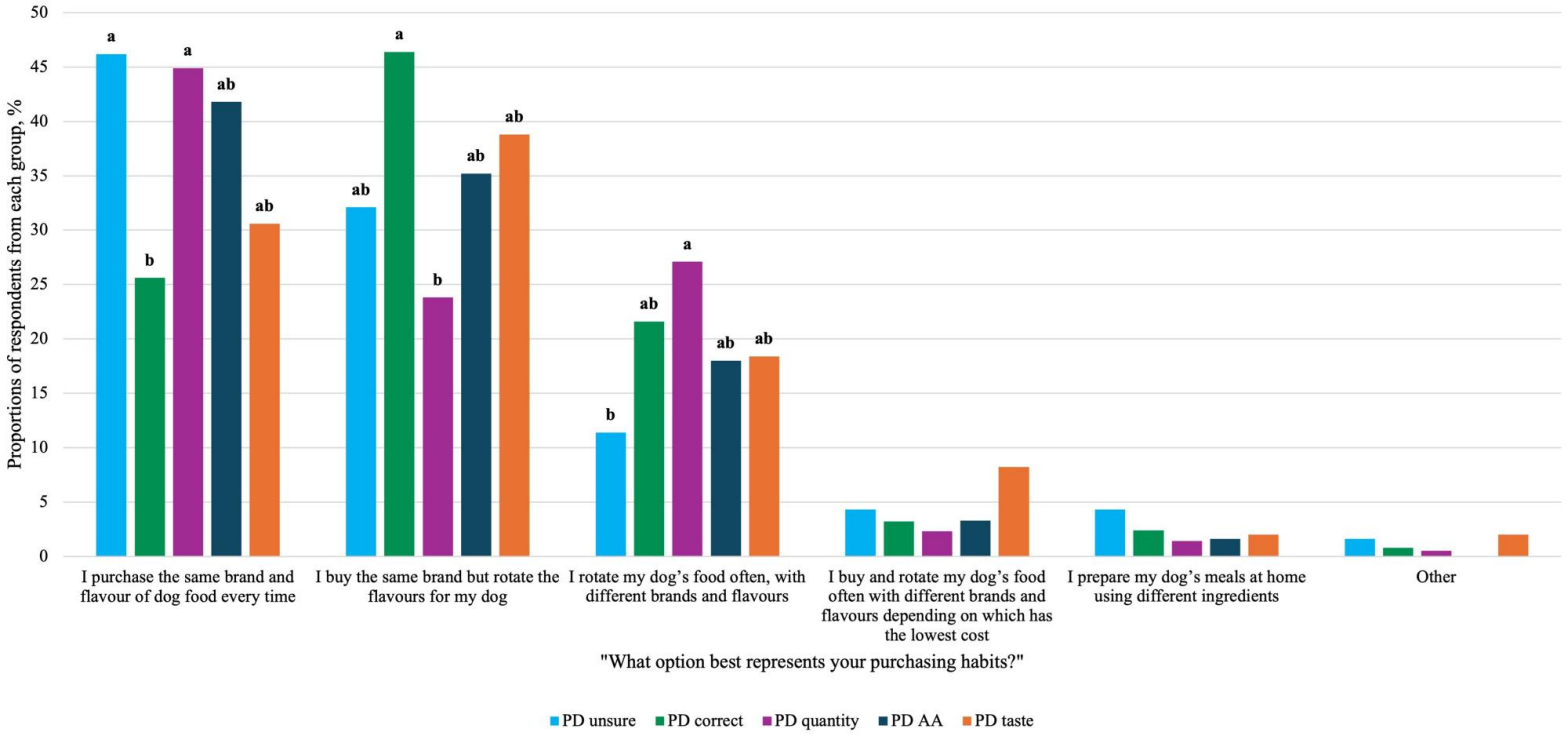
Dog food purchasing habits of respondents from different knowledge groups (*n* = 691). Knowledge groups in the same category that do not share a common letter are significantly different at *P* < 0.05.

Across knowledge groups, a higher proportion of respondents in the PD quantity (44.9%) and PD unsure (46.2%) groups reported purchasing the same brand and flavor of dog food every time compared with the PD correct group (25.6%; *P* < 0.001); however, these proportions did not differ from those observed in the PD AA (41.7%) and PD taste (30.6%) groups (*P* > 0.05; Figure 3). Conversely, a greater proportion of respondents in the PD correct group (46.4%) reported rotating their dog’s food often across different brands and flavors compared with the PD quantity group (27.1%; *P* < 0.001), while proportions in the PD unsure (32.1%), PD AA (35.2%), and PD taste (30.6%) groups did not differ from either group (*P* > 0.05). Moreover, a greater proportion of respondents in the PD quantity group reported that they rotate their dog’s food often, with different brands and flavors (27.1%) compared with the PD unsure group (11.4%; *P* < 0.001). In contrast, this purchasing behavior did not differ among the PD correct (21.6%), PD AA (18.0%), and PD taste (18.4%) groups (*P* > 0.05).

When asked where respondents primarily buy their dog’s food, 44.8% reported they buy it from a grocery store, making it the most common choice. This was followed by pet stores (31.1%), online shopping (17.3%), veterinary clinics (0.6%), and 5.8% of respondents reported they buy it from somewhere other than what is listed above. Interestingly, a greater portion of respondents in the PD unsure (53.3%) and PD taste (53.1%) groups, primarily buy their dog’s food from a grocery store compared to PD quantity respondents (36.4%, *P* = 0.001). Conversely, 43.5% of respondents in the PD quantity group reported primarily purchasing their dog’s food at a pet store, compared to only 16.8% of respondents from the PD unsure group (16.8%, *P* = 0.001). There were no differences among these purchasing behaviors across other PQ knowledge groups (*P* > 0.05).

Later, respondents were asked to rate the impact of various claims on their decision to purchase dog food using a 5-point scale. Scores of 0 to 3 indicated no to neutral impact, while scores of 4 to 5 indicated that the claim had some influence on their purchasing decision. The claims below are listed in order from most to least frequently reported as having an impact: excellent protein sources (72.3%), high-quality protein (68.6%), protein #1 ingredient (62.2%), HP diet (59.2%), animal meat 1st ingredient (58.6%), 100% traceable ingredients (53.6%), contains fresh meat (52.9%), humanely raised animals (49.1%), responsibly sourced ingredients (i.e. cage-free eggs) (45.5%), no animal by-product (41.4%), sustainably grown crops (40.1%), no pulse ingredients (32.6%), and raw-meat-based (31.8%).

**Figure 4:**
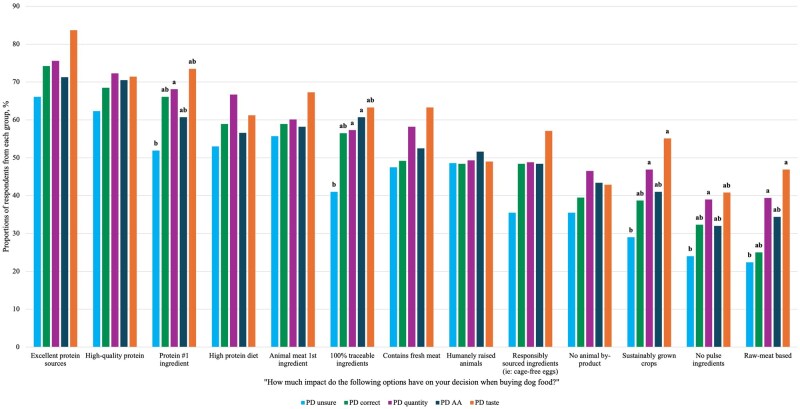
The impact that claims on dog food have on the purchasing decisions of respondents from different knowledge groups (*n* = 691). Knowledge groups in the same category that do not share a common letter are significantly different at *P* < 0.05. Respondents could select all options that applied.

More respondents in the PD taste (46.9%) and PD quantity (39.4%) knowledge groups reported that a “raw-meat-based” claim had an impact on purchasing decisions compared to only 22.4% of people who selected that they were unsure about how to define PQ (*P* < 0.001), but these groups were not different from PD AA (34.4%) or PD correct (25.0%; Figure 4). Moreover, the majority of respondents in the PD quantity (68.1%) group reported that “Protein #1 ingredient” had an impact on purchasing decisions compared to people who are unsure about how to define PQ (51.9%; *P* = 0.005). The proportions of both groups do not differ from those of the PD taste group (73.5%), PD correct (66.1%) group, or PD AA group (60.7%; Figure 4). Additionally, 39.0% of respondents from PD quantity reported that a “No pulse ingredients” claim influenced their purchasing decision, compared to 24.0% of PD unsure (*P* = 0.021), while similar proportions were observed among PD taste (40.8%), PD correct (32.3%), and PD AA (32.0%; Figure 4). Greater proportions of PD taste (55.1%) and PD quantity (46.9%) reported that a “Sustainably Grown Crops” (55.1%) claim impacted their purchasing decision compared to PD unsure (29%; *P* = 0.001), but all groups were similar to PD AA (41.0%) and PD correct (38.7%; Figure 4). Moreover, a greater portion of the PD quantity (57.3%) and PD AA (60.7%) groups reported a “100% Traceable Ingredients” claim had an impact on purchasing decisions compared to the PD unsure (41%; *P* = 0.002) but were not different than proportions of PD taste (63.3%) and PD correct (56.5%; Figure 4).

For the statement “I check the amount of protein in my dog’s diet” 39.8% of respondents reported that the statement describes them, while 34.6% indicated it does not describe them, and 25.6% expressed neutrality. There was a positive correlation (*r* = 0.2528) between people who reported tracking their protein intake in some capacity and respondents checking the protein amount in their dog’s diet (*P* < 0.001).

Respondents could select multiple options when reporting the challenges they face when purchasing dog food. The most frequently reported challenges were high cost (50.2%), followed by too many filler ingredients (32.6%) and unfamiliar ingredients (32.4%). Other challenges were their dog not liking the taste (31.0%), too many options (22.4%), limited product availability (19.1%), not enough protein (17.5%), too many claims (14.0%), presence of allergens (11.9%), convenience of use (5.2%), too much protein (4.1%), or other concerns (1.6%). Approximately 10.9% of respondents reported that they face few to no challenges when purchasing dog food.

### Dog owner’s acceptance of alternative protein sources in dog food

**Figure 5:**
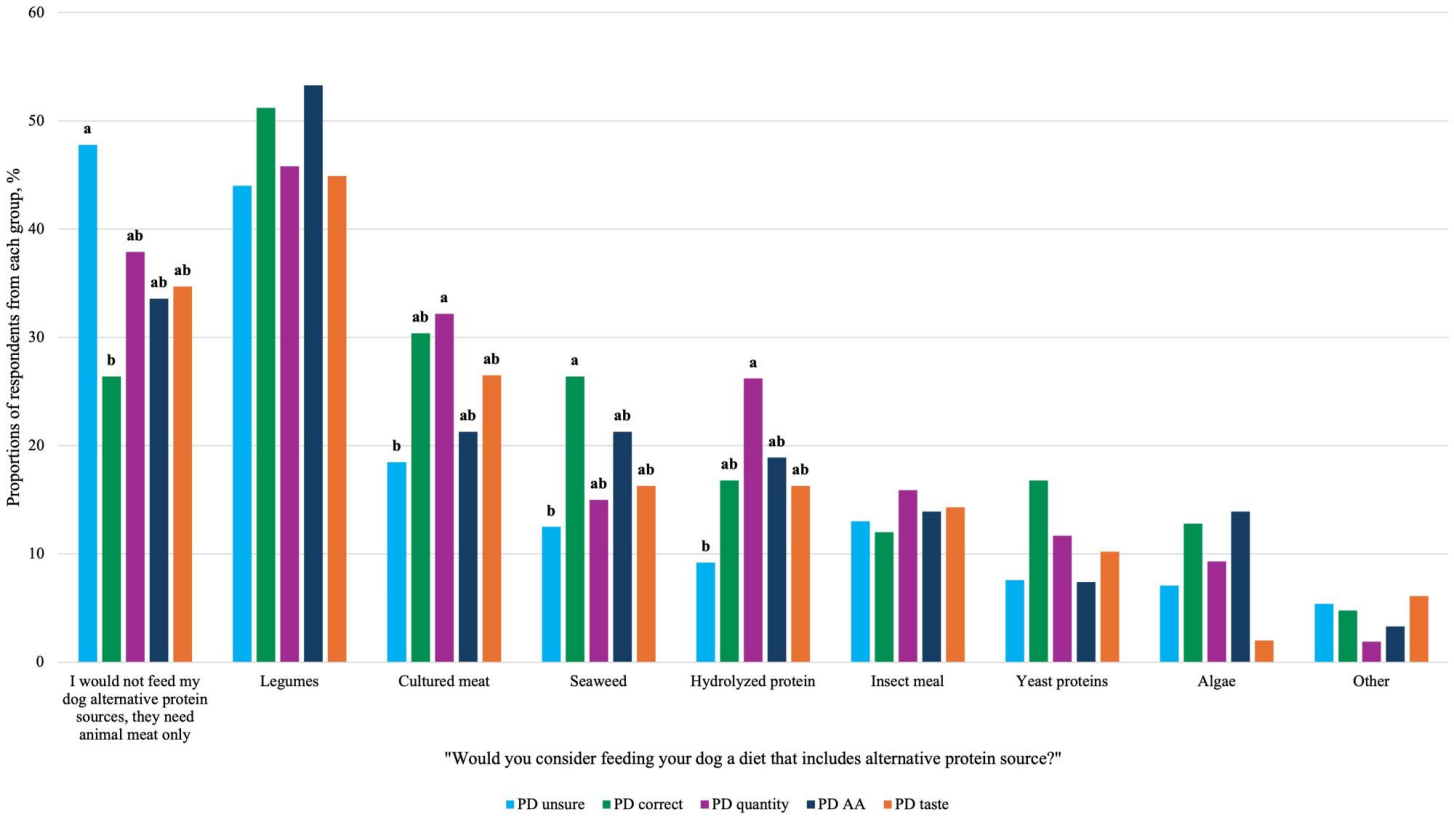
Proportion of different knowledge groups reporting which alternative protein sources they would accept in their dog’s diet (*n* = 691). Proportions in the same category that do not share a common letter are significantly different at *P* < 0.05. Respondents could select all options that applied.

From the list provided, the most commonly accepted alternative protein source by dog owners was legumes (soy, chickpeas, peas, lentils, etc.), with 47.8% of respondents open to feeding their dogs a diet that is legume-inclusive. However, 37.6% of respondents stated that they would not feed their dog alternative protein sources, believing their dog needs animal meat only. Meanwhile, other notable alternative protein sources that respondents reported they would consider feeding their dog include cultured meat (26.0%), hydrolyzed protein (18.1%), seaweed (17.7%), insect meal (14.0%), yeast proteins (10.7%), and algae (9.7%). Between the knowledge groups, a greater portion of the PD unsure group (47.8%) reported that they would not feed their dog a diet with alternative protein sources compared with the PD quantity (37.9%) and PD correct groups (26.4%; *P* = 0.016), but these groups were not different from PD taste (34.7%) or PD AA (33.6%; *P* > 0.05). Similar proportions across knowledge groups reported willingness to feed diets containing legumes, insect meal, yeast protein, algae, or other alternative protein sources (*P* > 0.05; Figure 5). In contrast, a greater proportion of the PD quantity group reported willingness to feed cultured meat (32.2%) and hydrolyzed proteins (26.2%) compared with the PD unsure group (18.5% and 9.2%, respectively; *P* < 0.001), while these proportions were similar to those of the other knowledge groups. Additionally, a greater proportion of the PD correct group reported willingness to feed seaweed (26.2%) compared with the PD unsure group (12.5%; *P* < 0.001); however, this did not differ from the other knowledge groups (Figure 5).

### Dog owners’ perception of protein importance in dog diets

When asked why they believe protein is important in their dog’s diet, respondents were able to select as many options as relevant to them. The most frequently reported reason was that protein was important to support their dog’s activity (78.1%). This was followed by supporting lean muscle (70.3%), immune function (68.2%), healthy skin and coat (56.5%), and satisfying hunger (36.5%). Only 2.3% of respondents indicated they do not believe protein is important in their dog’s diet.

Notably, there were significant differences between those who reported feeding a HP diet and the reported activity levels of their dogs. Among respondents whose dogs received only 0–15 min of daily activity, a greater proportion did not feed a high-protein diet (15.9%) compared to those who did (5.8%; *P* < 0.001). Conversely, among those whose dogs received 30–60 min of exercise per day, 36.3% fed a high-protein diet, compared to 28.5% who did not (*P* < 0.001). Similarly, among dogs that exercised more than 90 min daily, 12.2% were fed a high-protein diet, versus only 6.0% who were not (*P* < 0.001). However, for dogs exercised 15–30 min or 60–90 min daily, there were no differences in HP feeding between groups.

Similarly, there were differences in HP feeding based on how respondents described their dog’s activity level. A greater proportion of those who described their dog as a “couch potato” did not feed a HP diet (8.7%) compared to those who did (4.2%). In contrast, more respondents who described their dog as “very active” (40.4%) or “an athlete” (5.0%) reported feeding a HP diet compared to those who did not (24.6% and 1.8%, respectively; *P* < 0.001).

When asked to rate the validity of the statement, “The amount of protein in the diet is more important than the source of protein, given it meets nutrient requirements,” 49.6% viewed it as true, 30.5% were neutral, and 19.4% viewed it as false. Interestingly, a greater proportion of PD correct (57.3%) and PD quantity (56.3%) believe that statement to be true compared to the proportion from the PD unsure (37.7%) group (*P* < 0.001), while all were similar to PD AA (48.4%) and PD taste (51.0%).

### DCE questions and WTP

#### Baseline model

The results of the baseline model are displayed in column 1 of Table 6. The benchmark product was chosen to be peas with low protein (20%) since they generated the lowest utility, meaning that they influenced choice the least. As expected, price was negatively associated with choice, such that as the price increased, the same product became less desirable (*P* < 0.001). The ASC was significant (*P* < 0.001), meaning that respondents find value in making a choice over opting for “prefer not to choose.” Of the predictor variables, all protein characteristics were positively associated with choice. Among them, chicken had the greatest influence on choice (*P* < 0.001), followed by chicken meal (*P* < 0.001) and then HP (*P* < 0.001).

**Table 6:** Multinomial logistic regression estimates for the baseline model and the protein level model.

	(1)	(2)
	Base model	Protein level interaction model
**ASC** ^1^	2.277***	2.308***
	(0.114)	(0.125)
**Chicken**	1.126***	1.137***
	(0.037)	(0.063)
**Chicken meal**	0.540***	0.448***
	(0.035)	(0.073)
**High protein**	0.121***	
	(0.027)	
High protein * peas		0.077
		(0.070)
High protein * chicken		0.050
		(0.071)
High protein * chicken meal		0.246***
		(0.070)
**Price**	−0.014***	−0.014***
	(0.001)	(0.001)
***N***	24012	24012
**Log-likelihood**	−6929.212	−6927.237
**χ^2^ (model significance)**	2746.447	2744.553
***P*-value**	0.000	0.000

*Notes*: Non-interaction variables are compared to the benchmark of peas with low protein.

1 ASC values are positive and significant. ASC indicates a general tendency for respondents to select a product rather than opt out, suggesting that the choice task was interpreted and completed as intended.

*** represents significance at 0.001 level. Standard errors are displayed in brackets within the same column, in the row below.

The WTP associated with the protein characteristics compared to the benchmark product (peas with low protein) are displayed in Table S1. Respondents were willing to pay approximately 103% more for a product containing chicken (*P* < 0.001), ∼48% more for an option with chicken meal (*P* < 0.001), and ∼11% more for a product with HP (*P* < 0.001), compared to the benchmark product (i.e. peas with low protein) at $80, respectively.

#### Protein level interaction model

Table 6 presents the results of a model that includes the interactions of protein level and protein sources. The interaction of HP was not significant on the choice of peas (*P* = 0.275) or chicken (*P* = 0.480), but was positive and significant for chicken meal (*P* < 0.001). However, the WTP was not affected by the interaction with HP for chicken meal, chicken or peas (*P* > 0.05).

#### PQ knowledge models: PD correct interaction and PD quantity interaction

In the PD correct model, PD correct is a dummy variable that equals one if the respondent correctly identified the definition of PQ as “the ability of an ingredient to meet the amino acid requirements of an individual” and zero otherwise. In the PD quantity model, PD quantity is a dummy variable that equals one if the respondent defined PQ as “protein quality is the total amount of protein in an ingredient, such that a higher quantity of protein means better quality,” and zero otherwise.

Collectively, the PD correct model and the PD quantity model are referred to as the Protein Quality Knowledge Models, and model estimation of the results is presented in Table 7. The PD correct model results were similar to the baseline model, such that chicken, chicken meal and HP had a positive influence on choice compared to the benchmark group (*P* < 0.001). However, the interactions of PD correct with chicken, chicken meal, and peas were all positive, suggesting they had a greater influence on choice for people who could correctly define PQ (all three interaction terms have *P* < 0.001). As such, the interaction with PD correct increased the WTP for all protein sources; in quantitative terms, respondents who correctly define PQ were willing to pay ∼40% more for chicken (*P* = 0.001), ∼50% more for chicken meal (*P* < 0.001), and ∼40% more for peas (*P* < 0.001) compared to respondents who could not correctly define PQ. However, HP negatively influenced choice for PD correct (*P* = 0.025), translating to the WTP of this group being ∼13% less compared to respondents who did not correctly define PQ (*P* = 0.027). This implies that when individuals can accurately define PQ, the amount of protein (HP) is less influential in their decision-making than the source of the protein.

**Table 7:** Multinomial logistic regression estimates for Protein Quality Knowledge Model.

	(1)	(6)	(7)
	Baseline model	PD correct interaction model	PD quantity interaction model
**ASC^1^**	2.277***	2.098***	
	(0.114)	−0.121	
**Chicken**	1.126***	1.136***	1.110***
	(0.037)	(0.041)	(0.044)
Chicken * PD_Correct		0.441***	
		(0.126)	
Chicken * PD_Quantity			0.253*
			(0.100)
**Chicken meal**	0.540***	0.529***	0.439***
	(0.035)	(0.039)	(0.042)
Chicken meal * PD_Correct		0.559***	
		(0.134)	
Chicken meal * PD_Quantity			0.525***
			(0.108)
Peas * PD_Correct		0.499***	
		(0.134)	
Peas * PD_Quantity			0.197
			(0.109)
**High Protein**	0.121***	0.149***	0.102**
	(0.027)	(0.030)	(0.032)
High Protein* PD_Correct		−0.152*	
		(0.068)	
High Protein * PD_Quantity			0.066
			(0.057)
**Price**	−0.014***	−0.014***	−0.014***
	(0.001)	(0.001)	(0.001)
***N***	24012	24012	24012
**Log-likelihood**	−6929.212	919.136	908.354
**χ^2^ (model significance)**	2746.447	740.330	756.614
***P*-value**	0.000	0.000	0.000

*Notes*: PD correct is a dummy variable that equals 1 if the respondent correctly defined protein quality (PQ) as “the ability of an ingredient to meet the amino acid requirements of an individual” and zero otherwise. PD quantity is a dummy variable that equals 1 if the respondent defined PQ as “the total amount of protein in an ingredient, such that a higher quantity of protein means better quality” and zero otherwise. Non-interaction variables are compared to the benchmark of peas with low protein.

1 ASC values are positive and significant. ASC indicates a general tendency for respondents to select a product rather than opt out, suggesting that the choice task was interpreted and completed as intended.

***, **, and * represent significance at 0.001, 0.01, and 0.05 levels, respectively. Standard errors are displayed in brackets within the same column, in the row below.

For respondents who equated PQ with quantity, the interaction of PD quantity and chicken (*P* = 0.012) and chicken meal (*P* < 0.001) positively influenced choice. This resulted in the PD quantity group being willing to pay ∼22% more for a product with chicken (*P* = 0.014) and ∼47% more for a product with chicken meal (*P* < 0.001) compared to respondents who selected a different definition for PQ. Meanwhile, the influence of the interaction between peas and HP was similar to other knowledge groups (*P* > 0.05).

## Discussion

American dog owners’ perceptions regarding PQ of dietary sources for themselves are reflected in how they think about their dog’s nutrition. This allows us to consider how people may purchase dog food based on their own habits and practices. Notably, irrespective of their own protein intake, respondents generally believe their dogs require “high protein” compared to themselves, a metric that is not currently defined. This belief likely stems from the perception that greater protein quantity indicates superior quality, in combination with the generalization that their dogs are carnivores due to their ancestry to wolves (Bosch et al. 2015). However, as hypothesized and elucidated through the DCE questions, in the absence of protein-related claims, the source of protein had the greatest influence on choosing dog food.

Consistent with economic theory, respondents preferred the lower-priced dog food when all other factors were equal, supporting the robustness of the present study (Mariel et al. 2021). Across all models, protein source had a stronger influence on choice than the amount of protein. Chicken was the most influential attribute across all models and contributed to the highest WTP. In line with previous findings by Simonsen et al. (2014), the origin of ingredients has strong effects on choice, as animal-based proteins and natural ingredients were rated higher than plant-based ones. Americans are more likely to consume a diet with reduced meat than a vegetarian-based diet (Asher and Peters 2020). This preference for animal-based ingredients was reflected in the DCE model, as although chicken meal was less preferred than chicken, it had a greater influence on product choice than peas.

Half of the respondents in the current study agreed with the statement that the amount of protein is more important than source, even when nutrient requirements are met. Thus, we investigated whether higher protein content increased consumers’ willingness to choose a less favorable ingredient, such as peas. While the interaction between 35% protein and peas did not positively influence the choice of product, it did positively influence the choice of chicken meal product. Higher protein did not further increase the value of chicken for respondents, likely because it is already considered a high-quality ingredient and a “natural” ingredient sought by consumers (Simonsen et al. 2014; Román et al. 2017). Interestingly, dog owners appear to evaluate protein attributes sequentially, first considering the source, then the amount of protein. When a protein source was less preferred or perceived as lower value, such as chicken meal, interaction with higher protein content increased the value of the overall product. This suggests respondents first evaluate the protein source, and if the product is of interest but not fully compelling, greater protein content can enhance its value and increase the likelihood of selection. This pattern may offer a useful strategy for increasing the appeal of alternative protein sources. While most dog owners in the current study expressed an openness to feeding diets with alternative proteins, these products are not a top choice in practice.

In the knowledge models, it is important to note that protein source had the greatest influence on choice for both the PD correct and PD quantity groups. Interestingly, the 35% protein diet was negatively associated with choice for the PD correct group compared to all other knowledge groups, while the PD quantity group valued the 35% and 20% protein diets similarly. This suggests a knowledge gap; once presented with the minimum recommended protein levels for dogs, both 20% and 35% may have been interpreted as “high protein” in the absence of claims. Overall, individuals who could correctly define PQ as the ability of an ingredient to meet the indispensable AA requirements of an individual were more likely to prioritize protein source when selecting dog food and to be open to alternative protein ingredients. Meanwhile, respondents in the PD quantity group did not place greater value on a 35% protein diet over a 20% protein diet, elucidating the implications of educating consumers on their dog’s needs on purchasing decisions.

Overall, the feeding formats and purchasing habits in the present study are similar to previously reported habits of American dog owners (O’Brien et al. 2024). In the current study, those individuals able to correctly define PQ (PD correct) tended to rotate between dog food flavors and brands, unlike those who believed that more protein is better or were unsure, who more often purchased the same product each time. Purchasing behavior of the respondents who could not correctly define PQ was similar to previous findings that dog owners are inclined to buy from a particular brand (Tesfom and Birch 2010). However, this behavior suggests that individuals with greater knowledge are more likely to diversify their choices within the brand. Rotation of commercial kibble can be a beneficial practice, possibly helping balance any digestibility or nutritional implications of one diet. Natural and processed ingredients differ in nutrient delivery due to variations in agricultural feed practices (te Pas et al. 2021) and the growing conditions of plant crops (Scharff et al. 2022). Therefore, rotating ingredients can help create complementary and complete nutrient profiles. Processing also affects the nutrient bioavailability in ingredients. For example, chicken meal can have variable bioavailability of its AAs depending on the batch (Crosbie et al. 2024). This variability does not diminish the value of chicken meal but rather highlights the importance of analyzing the overall nutrient composition after processing and using a variety of ingredients to help balance the final AA profile of the diet. Similarly, human dietary guidelines generally promote diversity of protein sources from plant and animal sources to meet protein and AA requirements. Diversifying complete diets or looking at overall nutrient composition in dog foods can mimic the feeding behavior of humans while maintaining the convenience of a complete diet format and also allows for the use of various ingredients to meet the needs of animals with consideration of the environmental and economic constraints of global food supply. Overall, this data should be interpreted with caution, as conclusions are based on respondents’ limited knowledge. Nevertheless, the findings highlight the importance of PQ knowledge in promoting more diverse choices and a better understanding of their dog’s nutrition.

As hypothesized, opinions regarding the quality of an ingredient as a protein source were translated from human to dog diets, suggesting that respondents believe what is good for them is likely good for their dog (anthropomorphism). While only 11% of dog owners follow a “high protein” diet themselves, approximately 50% of dog owners reported feeding a “high protein” diet to their dog, and around 60% reported that a “high protein” claim highly impacted their decision to buy dog food. Moreover, in the current study, 62% of respondents reported looking for “protein #1 ingredient” claims on their dog food, which is double the number of Americans who reported looking for this label just three years ago (Banton et al. 2021). Antoniak et al. (2022) report that a “high protein” nutrition claim on meat patties increased consumer willingness to consume a product, and consumers also believed the product to be of better quality when compared to the same product without this claim. The top two claims reported to have an impact on purchasing decisions in the current study were “excellent protein sources” and “high-quality protein,” closely followed by a “high protein” claim, reinforcing the notion that dog owners believe quantity is directly related to quality. This belief can be misleading and can contribute to overconsumption due to perceived health benefits (Kaur et al. 2017). It can also suggest a false narrative for pet food manufacturers that consumers prefer greater amounts of protein in their dogs’ diet. While 39% of respondents in the current study reported checking the amount of protein in their dog’s diet, it remains unclear what they consider a “high” protein amount, whether they associate specific values with a “high protein” claim, or if the claim alone shapes their perception due to its link with perceived quality. This potentially creates a gap between what owners believe they are feeding and what manufacturers aim to deliver.

High-protein diets are expensive to formulate due to the cost of protein ingredients, and they also place additional pressure on global ingredient supplies and the environment. Dietary protein that is not utilized by the dog is metabolized and excreted as nitrogen waste into the environment. Although estimating nitrogen waste from pets is challenging, recent global estimates suggest that dogs excrete approximately 3.82 million tonnes of nitrogen annually through urine and feces (Cowan et al. 2024). Excess nitrogen can have detrimental environmental effects, such as eutrophication of water systems (Follett and Hatfield, 2001; Malone and Newton, 2020). Optimizing protein delivery can therefore improve nutrient utilization in dogs while reducing environmental waste. Moreover, the production of pet food itself has implications for land use and greenhouse gas emissions. Diversity of protein ingredients, with plant or alternative sources, can help reduce the overall environmental implications as well. In an analysis of 31 dry dog foods available in the United Kingdom, Brociek and Gardner (2025) report that plant-based diets had the lowest environmental impact across land use, greenhouse gas emissions, and eutrophication compared to diets containing poultry, beef, or lamb. While it is unlikely that all consumers will accept solely plant-based diets for their dogs, formulating diets with mixed plant and animal ingredients can help maintain the desired protein content, quality, and palatability of pet food while reducing strain on the environment and global ingredient supply (Kaplan and McClements 2025).

Over the past two decades, dog owners have increasingly shifted from relying primarily on veterinarians for nutrition advice to seeking information online. In 2008, over 60% of U.S. dog owners reported veterinarians as their primary source of pet nutrition information compared with only 7% using online sources (Laflamme et al. 2008). By 2018, reliance on veterinarians decreased to 40%, while online sources increased to 24% (Schleicher et al. 2019). In the present study, more than 50% of respondents use online sources for pet food information, while also maintaining reliance on their veterinarian for nutritional guidance. With the rise of online sources, dog owners are exposed to an overwhelming and often conflicting volume of information that is frequently presented as being personalized for unique individual needs yet lacks empirical evidence. This information overload makes it difficult for dog owners to evaluate accuracy or relevance (Benselin and Ragsdell 2016; reviewed in Misuraca et al. 2024), often reinforcing simplified interpretations like “more protein is better.” As a result, marketing claims such as “high protein” gain disproportionate influence, despite unclear nutritional significance. While dog owners appear to seek PQ over quantity, there is an evident need for education on what contributes to PQ. Public knowledge and perceptions are key drivers of purchasing behavior (Biel and Grankvist 2001), a relationship that is further amplified by internet-driven normalization of specific nutrition ideologies. In human nutrition, standardized metrics such as the Digestible Indispensable Amino Acid Score (DIAAS) have been developed to evaluate PQ (FAO 2013), and similar metrics can be applied to pet food. Recently, DIAAS-like scores have been calculated for several common animal and plant-based protein ingredients used in pet food, using dog-specific AA requirement reference patterns (Templeman and Shoveller 2022). Given that pet food is typically marketed as complete diets, PQ metrics should reflect the overall nutritional quality of the product, rather than individual ingredients. Marketing claims are meant to educate the consumer and guide purchasing decisions, therefore, standardized protein claims could help the pet food industry educate owners, promote consistency in PQ across the market, and improve product navigation by using terminology already familiar from human nutrition.

A limitation in this study is that dog owners were not asked to quantify the amount of protein they would consider as “high protein,” nor is this defined in the pet food industry. Moreover, differences in PD knowledge group sizes limit direct comparisons of behaviors and perceptions. In particular, the small sample sizes for the PD taste and AA groups resulted in larger standard errors and wider confidence intervals, thereby reducing statistical power for pairwise comparisons. Additionally, there are limitations to using WTP measures, as individuals often overestimate their WTP in hypothetical scenarios, a phenomenon known as hypothetical bias, which can undermine the validity of DCE responses (Champ et al. 2009). To mitigate hypothetical bias, a cheap talk script was included, offering participants more context and thereby improving the robustness of the results (List 2001; Murphy and Stevens 2005). Nonetheless, the results offer comparative insight into ingredient valuation in a commercial context.

The information regarding dietary protein often presents a confusing contrast between its recognized importance and an overwhelming amount of sometimes contradictory messages, leaving consumers unclear about what is good, bad, and appropriate for their pets. Ultimately, consumers gravitate toward what they recognize. This study elucidates that dog owners find greater value in the source of protein they feed their dogs, with little knowledge of how much protein their dogs get and minimal understanding of PQ. To support informed purchasing decisions, greater consumer education and standardized marketing practices are essential. These efforts would help align product offerings with dogs’ actual nutritional needs, steering the industry away from excessive or imbalanced formulations and toward balanced and conservative formulation prioritizing optimal nutrient delivery and decreasing harmful waste to the environment.

## Supplementary Material

skag136_Supplementary_Data

## Data Availability

The data underlying this article can be shared on reasonable request to the corresponding author.

## Supplementary data

*Conflict of interest statement.* P.S. is funded by Champion Petfoods through an NSERC Alliance and Mitacs grant and is an intern as part of the Mitacs Accelerate program with Champion Petfoods. C.P.F.M is an employee of Protein Industries Canada and a former employee of Pulse Canada and Kellogg Canada. A.K.S. has received honoraria and research funding from various pet food manufacturers and ingredient suppliers in addition to provincial and federal granting agencies and declares that she serves on the Trouw Nutrition and Champion Petfoods Scientific Board. S.B, Z.P, M.vM, and G.B do not have any conflicts of interest.

## Abbreviations

AA: amino acids
ASC: alternative-specific constant
AAFCO: Association of American Feed Control Officials
CP: crude protein
DCE: discrete choice experiment
DIAAS: digestible indispensable amino acid score
IAAO: indicator amino acid oxidation
PDCAAS: protein digestibility corrected amino acid score
PD: protein definition
RDA: recommended dietary allowance
USA: United States of America
WTP: willingness to pay
