# Supplementary material for: Does quantity equal quality? Source of protein influences American dog owner purchasing decisions more than the quantity of protein in the absence of marketing claims
Source: J Anim Sci. 2026 May 8;104:skag136. doi: 10.1093/jas/skag136 (PMC13201265; doi:10.1093/jas/skag136)
Supplement: skag136_Supplementary_Data [file skag136_supplementary_data.zip › SupplementaryTable_1.docx]

**Supplementary Table 1: Willingness to pay for dog food based on the influence of different dietary protein attributes, in choice task questions.**

| **Variable** | WTP* | SE | T-value | P- value | 95% CI |
| --- | --- | --- | --- | --- | --- |
| **(1) Base Model** |  |  |  |  |  |
| Chicken | 82.032 | 6.781 | 12.098 | 0.000 | [68.742, 95.322] |
| Chicken Meal | 39.369 | 4.032 | 9.763 | 0.000 | [31.466, 47.273] |
| High Protein | 8.853 | 1.928 | 4.591 | 0.000 | [5.074, 12.633] |
|  |  |  |  |  |  |
| **(2) High Protein Model** |  |  |  |  |  |
| Chicken | 82.098 | 8.130 | 10.098 | 0.000 | [66.164, 98.032] |
| Chicken Meal | 32.361 | 6.132 | 5.278 | 0.000 | [20.343, 44.378] |
| High Protein | 17.724 | 5.010 | 3.538 | 0.000 | [7.905, 27.543] |
| High Protein * Chicken | -1.929 | 8.382 | -0.230 | 0.818 | [18.357, 14.498] |
| High Protein * Chicken Meal | 12.197 | 8.189 | -1.489 | 0.136 | [-3.854, 28.248] |
|  |  |  |  |  |  |
| **(6) Knowledge Model** |  |  |  |  |  |
| Chicken | 81.199 | 7.182 | 11.305 | 0.000 | [67.122, 95.276] |
| Chicken * PQ_Correct | 40.782 | 10.073 | 4.049 | 0.000 | [21.039, 60.525] |
| Chicken * PQ_Quantity | 27.255 | 7.876 | 3.461 | 0.001 | [11.818, 42.692] |
| Chicken meal | 27.786 | 4.180 | 6.648 | 0.000 | [19.593, 35.978] |
| Chicken meal * PQ_Correct | 58.709 | 11.294 | 5.198 | 0.000 | [36.572, 80.845] |
| Chicken meal * PQ_Quantity | 51.741 | 9.246 | 5.596 | 0.000 | [33.620, 69.863] |
| Peas * PQ_Correct | 43.737 | 10.776 | 4.059 | 0.000 | [22.617, 64.856] |
| Peas * PQ_Quantity | 23.886 | 8.423 | 2.836 | 0.005 | [7.377, 40.395] |
| High Protein | 10.241 | 2.708 | 3.781 | 0.000 | [4.933, 15.548] |
| High Protein* PQ_Correct | -10.399 | 5.255 | -1.979 | 0.048 | [-20.699, -0.099] |
| High Protein * PQ_Quantity | 2.003 | 4.350 | 0.460 | 0.645 | [-6.523, 10.529] |

*WTP = willingness to pay presented in dollar amount relative to the monetary values within the choice tasks ($80, $95, $110).

Notes: *High Protein* is a dummy variable that equaled one if the respondent selected the option with 35% protein and zero otherwise. *Chicken* is a dummy variable that equaled one if the respondent selected the option with Chicken and zero otherwise. *Chicken Meal* is a dummy variable that equaled one if the respondent selected the option with Chicken Meal and zero otherwise. *Peas* is a dummy variable that equaled one if the respondent selected the option with Peas and zero otherwise.

Non-interaction variables are compared to the benchmark of peas with low protein.

*PD correct* is a dummy variable that equals 1 if the respondent correctly defined protein quality (PQ) as “the ability of an ingredient to meet the amino acid requirements of an individual” and zero otherwise. *PD quantity* is a dummy variable that equals 1 if the respondent defined PQ as “the total amount of protein in an ingredient, such that a higher quantity of protein means better quality” and zero otherwise.
