# Supplementary material for: Does quantity equal quality? Source of protein influences American dog owner purchasing decisions more than the quantity of protein in the absence of marketing claims
Source: J Anim Sci. 2026 May 8;104:skag136. doi: 10.1093/jas/skag136 (PMC13201265; doi:10.1093/jas/skag136)
Supplement: skag136_Supplementary_Data [file skag136_supplementary_data.zip › Supplementary_finalconsumersurvey.pdf]

Consumer Perceptions on Protein Quality in Dog Food

Start of Block: Consent form

CONSENT FORM Consumer and Professional Perceptions on Protein Quality in Dog Food survey - You are invited to participate in this survey conducted by faculty at the University of Guelph, Department of Animal BioSciences. The purpose of this form is to provide you with the information needed to make an informed decision about participating in this research. The Researchers Principal Investigator: Anna Kate Shoveller, Department of Animal BioSciences, University of Guelph, ashovell@uoguelph.ca, 519-824-4120 ext. 53140 Pawanpreet Singh, Department of Animal BioSciences, University of Guelph, pawanpre@uoguelph.ca Sydney Banton, Department of Animal BioSciences, bantons@uoguelph.ca Other Investigators: Michael von Massow, Department of Food, Agricultural and Resource Economics, University of Guelph, mvonmass@uoguelph.ca Purpose of the study: The objective of this study is to survey the perspective of dog owners and pet professionals (veterinary professionals, breeders and pet store employees), to understand the current level of knowledge and opinions on dietary protein for pets. This will help build the language and tools to help pet owners and professionals navigate adequate dietary protein choices for their dogs. We will ask questions about you, your dog, and your choices when purchasing/advising pet food. This project is sponsored by Mitacs Accelerate–NSERC Alliance, alongside Champion Petfoods Holdings Inc. The data collected from the survey will be used as part of a PhD Thesis at the University of Guelph, published in an academic journal, and may be presented as an oral or poster presentation. Champion Petfoods Inc. may use this data for business plans; however, no personal identifying information will be available to them or the researchers to maintain the privacy of all participants. Procedures: If you agree to participate in this study, we would ask you to do the following things: Completing the survey should take no more than 15 minutes, however, there are no time restrictions and you may take as long as you like to complete the survey. If you respond to this survey using a public computer, we recommend you take the following precautions upon completion of the survey to clear all private data from the computer you are using to respond to the survey: 1. Clear the browsing history 2. Clear the cache 3. Clear the cookies 4. Clear the authenticated session 5. LOG OFF If you are using Internet Explorer, the first 4 steps can be accomplished by going to Tools and selecting Delete Browser History. Your browser application may have a similar system to remove potentially identifying personal information. Risks and Benefits: There are minimal risks involved when participating in this survey, such as possibly uncomfortable questions related to income, gender and/or personal dietary/exercise habits. If at any point during the survey you do not feel comfortable answering a question, please exit the survey. If you withdraw from the survey, all of your data will be deleted. The survey is anonymous and does not collect any identifying information. By participating in this survey, you are contributing to research that will be used to improve the

understanding and language used to help dog owners make decisions when purchasing pet food and highlight areas that the pet food industry can improve on to ensure improved quality and communication to pet owners. Once you submit the survey, you will receive the incentive described before entering into the survey. There is otherwise no direct benefit from completing the survey.   Participation and Withdrawal: You can choose whether to be in this study or not. If you begin this survey and choose to withdraw before completing it, all data will be deleted. The survey is anonymous and therefore not linked to your personal information. Once you submit your responses, you cannot withdraw, thus if at any point you wish not to share your data, please close the browser and the incomplete responses will not be used. The investigator may withdraw anonymous responses from this research if circumstances arise that warrant doing so. Rights of Research Participants: This project has been reviewed by the Research Ethics Board for compliance with federal guidelines for research involving human participants. You do not waive any legal rights by agreeing to take part in this study. If you have any questions regarding your rights and welfare as a research participant in this study (REB 24-02-001) please contact: Manager Research Ethics, University of Guelph, reb@uoguelph.ca, 519-824-4120 ext. 56606. Confidentiality: No individual identifiers will be attached to the survey data. After the survey has concluded, the raw data collected will only be available to the researchers associated with the University of Guelph: the primary investigator and the other investigators. This information will be stored on a password-protected computer for up to 18 months after the data has been published.   Exclusion Criteria: •   Relationship with researchers - no participants with a professional or personal relationship with the researchers will be allowed to participate in the research. •   Cannot work in pet food formulation   Inclusion Criteria •   Must be over 18 years of age • Must be a dog owner with at least one dog that is consuming a non-prescription diet and must be the primary person responsible or share responsibility for selecting your dog’s food • If not dog owner, must be a small animal veterinarian, dog breeder or work in pet food industry   If you have read the information provided for the study “Consumer and professional perceptions on protein quality in dog food” as described herein, and your questions have been answered to your satisfaction, please answer the following question: Do you agree to participate in the research outlined above?

○ Yes

○ No

End of Block: Consent form

Start of Block: Screening Questions

Q1 What is your age?

- Under 18 years
- 18-24 years
- 25-34 years
- 35-44 years
- 45-54 years
- 55-64 years
- 65+ years

Page Break

Q2 What is your sex?

- ◯ Female
- ◯ Male
- ◯ Intersex
- ◯ Prefer not to answer

Page Break

Q3 Please select if you work as one of the following:

- ○ Small animal veterinarian
- ○ Small animal veterinarian technician
- ○ Dog breeder
- ○ Pet store employee
- ○ Formulation or contributor to the development of commercial pet food
- ○ I do not work in any of the above professions

Page Break

Q4 Do you currently own any of the following pets? Select all that apply

- ▢ I do not have a pet
- ▢ Cat
- ▢ Dog
- ▢ Rabbit
- ▢ Fish
- ▢ Turtle
- ▢ Hamster
- ▢ Lizard
- ▢ Other

End of Block: Screening Questions

Start of Block: Screening Questions- Consumer Only

Q5 Is your dog on a therapeutic diet that you purchase from the vet?

- ○ Yes
- ○ No
- ○ I'm not sure

Page Break

Q6 Who in your household is primarily responsible for choosing your dog's food?

- ○ I am primarily responsible for choosing my dog's food
- ○ I share responsibility for choosing my dog's food equally with someone else
- ○ I am not at all responsible for choosing my dog's food

End of Block: Screening Questions- Consumer Only

Start of Block: Main Questions

Q7 What dietary option best describes your diet? Select all that apply

- ☐ Kosher
- ☐ Halal
- ☐ Grain-free
- ☐ Organic
- ☐ Omnivore (eats both animal meat/products and plants)
- ☐ Ovo-vegetarian (avoid animal meat and dairy, but consume eggs)
- ☐ Lacto-vegetarian (avoid animal meat and eggs, but consume dairy)
- ☐ Ovo-lacto vegetarian (avoid animal meat, but consume dairy and eggs)
- ☐ Pescatarian (eat seafood and plants, but avoid land-animal meat)
- ☐ Vegan
- ☐ Dairy-free
- ☐ Meat minimalist/flexitarian (cautiously limiting meat consumption)
- ☐ Raw
- ☐ Ketogenic
- ☐ High protein diet
- ☐ Diabetic diet
- ☐ Renal or kidney diet

- ☐ Gluten-free or celiac diet
- ☐ Low or carbohydrate-free diet
- ☐ Low fat/low-calorie diet
- ☐ Low sodium diet
- ☐ Low sugar or sugar-free diet
- ☐ Low fiber diet
- ☐ High fiber diet
- ☐ No processed foods (i.e. no additives, minimal ingredient diet)
- ☐ I do not follow a specific dietary pattern.
- ☐ Other

Page Break

Q8 If you selected vegetarian, vegan, or meat minimalist please select why you do not eat or reduce your consumption of animal-derived foods. Select all that apply.

- ☐ Religious
- ☐ Animal Welfare
- ☐ Environmental consideration
- ☐ Health/Nutrition
- ☐ Dietary restriction
- ☐ Taste preference
- ☐ Other

Page Break

Q9 Do you track your protein consumption?

○ I track my protein consumption in grams per day

○ I track the types of food I am eating to make sure I have protein in every meal

○ I track the types of food I am eating to make sure I consume protein in at least one meal daily

○ I do not consider or track how much protein I am eating

○ I am not sure what foods have protein

○ Other

Page Break

Q10 Regardless of personal dietary preference, rank the following ingredients, in terms of the quality of protein they provide in the human diet:

	Terrible (1)	Poor (2)	Average (3)	Good (4)	Excellent (5)
Chicken	○	○	○	○	○
Chicken by-products (liver, gizzards, etc.)	○	○	○	○	○
Peas	○	○	○	○	○
Tofu	○	○	○	○	○
Beans (i.e. chickpeas, black beans, kidney beans, navy beans, pinto beans)	○	○	○	○	○
Beef	○	○	○	○	○
Pork	○	○	○	○	○
Insects	○	○	○	○	○
Whey protein powder	○	○	○	○	○
Pea protein powder	○	○	○	○	○

Page Break

Q11 Which of the following options do you believe best defines “protein quality” of an ingredient:

- ○ Protein quality is the total amount of protein in an ingredient, such that a higher quantity of protein means better quality
- ○ Protein quality is the ability of an ingredient to meet the amino acid requirements of an individual
- ○ Protein quality is the total number of amino acids present in an ingredient, where the higher total amino acids means better quality
- ○ Protein quality is the taste and palatability of a protein-rich food, a higher protein food tastes better than a lower protein quality food.
- ○ I am not sure

Page Break

Q12 Please allocate 100 points between the following items in order of importance to your diet (i.e. - if you believe an item is more important, assign more points to it).
Vitamins : _______
Fats : _______
Proteins : _______
Minerals : _______
Carbohydrates (includes dietary fiber) : _______
Total : ________

End of Block: Main Questions

Start of Block: Dog questions

Q13 How many dogs do you own?

- ◯ 1
- ◯ 2
- ◯ 3
- ◯ 4+

Page Break

If you have more than one dog answer the following according to one dog, whose birthday is earliest in the year:

Q14 What type of dog do you own?

- ◯ Pure-bred dog
- ◯ Mixed-breed dog
- ◯ Not sure
- ◯ Other

Page Break

Q15 Is this your first dog?

- Yes
- No

Page Break

Q16 What is the size of your dog?

- ◯ Up to 8 lbs / 3.6 kg
- ◯ 8-22 lbs / 3.6-10 kg
- ◯ 22-55 lbs / 10-25 kg
- ◯ 55-100 lbs / 25-45.4 kg
- ◯ More than 100 lbs / 45.4 kg

Page Break

Q17 What is the sex of your dog?

- ○ Male
- ○ Neutered male
- ○ Female
- ○ Spayed Female

Page Break

Q18 Where did you get your dog?

- Breeder
- Animal shelter / rescue
- Friends/family
- Pet store
- Stray
- Other

Page Break

Q19 What option best represents your purchasing habits?

- ◯ I purchase the same brand and flavour of dog food every time
- ◯ I buy the same brand but rotate the flavours for my dog
- ◯ I rotate my dog’s food often, with different brands and flavours
- ◯ I buy and rotate my dog’s food often with different brands and flavours depending on which has the lowest cost.
- ◯ I prepare my dog’s meals at home using different ingredients
- ◯ Other

Page Break

Data quality is very important to us. To show that you are paying attention, please select "vegetarian".

- Omnivore
- Carnivore
- Vegetarian
- Vegan
- Meat Minimalist

Page Break

Q20 What are the biggest challenges you face when choosing a dog food? Select all that apply.

- ▢ Presence of allergens
- ▢ Too many unfamiliar ingredients
- ▢ Too many options
- ▢ My dog doesn’t like the taste
- ▢ Too many claims
- ▢ Confusion about which is best for my dog
- ▢ High cost
- ▢ Not enough protein
- ▢ Too much protein
- ▢ Convenience of use
- ▢ Product availability
- ▢ Too many filler ingredients
- ▢ No/few challenges
- ▢ Other

Page Break

Q21 From the options below, select what is most similar to what you are currently feeding your dog. Select all that apply i.e. if you mix kibble and canned food for your dog - select both.

- ☐ Kibble
- ☐ Raw
- ☐ Home cooked
- ☐ Gently cooked
- ☐ Freeze-dried
- ☐ Canned
- ☐ Other

Page Break

Q22 What claims best describe the food you currently feed? Select all that apply.

- ▢ Grain-free
- ▢ Grain-inclusive
- ▢ High protein
- ▢ Plant-based diet
- ▢ Hydrolyzed protein
- ▢ Limited ingredient
- ▢ No added supplements
- ▢ Other
- ▢ I do not look for claims

End of Block: Dog questions

Start of Block: Cheap Talk- Consumers

Cheap Talk- The products you are selecting may or may not be available for purchase today, but we are interested in what you think of these products. Please select the product you are most interested in. While the choices are hypothetical, please choose the foods as though you are purchasing these products. The minimum recommended amount of protein for adult dogs is 18%. Regardless of your dog's eating restrictions, please carefully read the following dog food options and select the product you are most interested in. The products are 30 lb bags of commercial dry dog food. Please note that the characteristics of the products that will vary include the protein ingredients (chicken, chicken meal and peas), the amount of protein, and the product’s price.

End of Block: Cheap Talk- Consumers

Start of Block: Choice Tasks

Choice Task 1: Please select the product you would most like to purchase

$USD per 30 lb bag of commercial dry dog food

**Attribute / Choice**	**Option 1**	**Option 2**	**Option 3**
1st 3 Ingredients	Chicken meal, rice, oats	Chicken, rice, oats	Prefer not to choose
Protein Percentage	35%	20%	—
Price	$95	$110	—
I would choose	○ Chicken meal (1)	○ Chicken (2)	○ Prefer not to choose (3)

Page Break

Choice Task 2: Please select the product you would most like to purchase

$USD per 30 lb bag of commercial dry dog food

Attribute / Choice	Option 1	Option 2	Option 3
1st 3 Ingredients	Chicken, rice, oats	Peas, rice, oats	Prefer not to choose
Protein Percentage	20%	35%	—
Price	$95	$110	—
I would choose	○ Chicken (1)	○ Peas (2)	○ Prefer not to choose (3)

Page Break

Choice Task 3: Please select the product you would most like to purchase

$USD per 30 lb bag of commercial dry dog food

**Attribute / Choice**	**Option 1**	**Option 2**	**Option 3**
1st 3 Ingredients	Chicken, rice, oats	Chicken meal, rice, oats	Prefer not to choose
Protein Percentage	20%	35%	—
Price	$95	$80	—
I would choose	○ Chicken (1)	○ Chicken meal (2)	○ Prefer not to choose (3)

Page Break

Choice Task 4: Please select the product you would most like to purchase

$USD per 30 lb bag of commercial dry dog food

**Attribute / Choice**	**Option 1**	**Option 2**	**Option 3**
1st 3 Ingredients	Chicken meal, rice, oats	Chicken, rice, oats	Prefer not to choose
Protein Percentage	35%	20%	—
Price	$110	$95	—
I would choose	○ Chicken meal (1)	○ Chicken (2)	○ Prefer not to choose (3)

Page Break

Choice Task 5: Please select the product you would most like to purchase

$USD per 30 lb bag of commercial dry dog food

Attribute / Choice	Option 1	Option 2	Option 3
1st 3 Ingredients	Peas, rice, oats	Chicken, rice, oats	Prefer not to choose
Protein Percentage	20%	35%	—
Price	$80	$95	—
I would choose	○ Peas (1)	○ Chicken (2)	○ Prefer not to choose (3)

Page Break

Choice Task 6: Please select the product you would most like to purchase

$USD per 30 lb bag of commercial dry dog food

**Attribute / Choice**	**Option 1**	**Option 2**	**Option 3**
1st 3 Ingredients	Peas, rice, oats	Chicken meal, rice, oats	Prefer not to choose
Protein Percentage	35%	20%	—
Price	$80	$95	—
I would choose	○ Peas (1)	○ Chicken meal (2)	○ Prefer not to choose (3)

Page Break

Choice Task 7: Please select the product you would most like to purchase

$USD per 30 lb bag of commercial dry dog food

**Attribute / Choice**	**Option 1**	**Option 2**	**Option 3**
1st 3 Ingredients	Chicken meal, rice, oats	Peas, rice, oats	Prefer not to choose
Protein Percentage	35%	20%	—
Price	$95	$80	—
I would choose	○ Chicken meal (1)	○ Peas (2)	○ Prefer not to choose (3)

Page Break

Choice Task 8: Please select the product you would most like to purchase

$USD per 30 lb bag of commercial dry dog food

**Attribute / Choice**	**Option 1**	**Option 2**	**Option 3**
1st 3 Ingredients	Chicken meal, rice, oats	Peas, rice, oats	Prefer not to choose
Protein Percentage	20%	35%	—
Price	$80	$110	—
I would choose	○ Chicken meal (1)	○ Peas (2)	○ Prefer not to choose (3)

Page Break

Choice Task 9: Please select the product you would most like to purchase
$USD per 30 lb bag of commercial dry dog food

Attribute /	Option 1	Option 2	Option 3
1st 3 Ingredients	Peas, rice, oats	Chicken, rice, oats	Prefer not to choose
Protein Percentage	20%	35%	—
Price	$80	$110	—
I would choose	○ Peas (1)	○ Chicken (2)	○ Prefer not to choose (3)

Page Break

Choice Task 10: Please select the product you would most like to purchase
$USD per 30 lb bag of commercial dry dog food

Attribute / Choice	Option 1	Option 2	Option 3
1st 3 Ingredients	Peas, rice, oats	Chicken, rice, oats	Prefer not to choose
Protein Percentage	35%	20%	—
Price	$80	$110	—
I would choose	○ Peas (1)	○ Chicken (2)	○ Prefer not to choose (3)

Page Break

Choice Task 11: Please select the product you would most like to purchase
$USD per 30 lb bag of commercial dry dog food

Attribute / Choice	Option 1	Option 2	Option 3
1st 3 Ingredients	Peas, rice, oats	Chicken meal, rice, oats	Prefer not to choose
Protein Percentage	20%	35%	—
Price	$80	$110	—
I would choose	○ Peas (1)	○ Chicken meal (2)	○ Prefer not to choose (3)

Page Break

Choice Task 12: Please select the product you would most like to purchase

$USD per 30 lb bag of commercial dry dog food

Attribute / Choice	Option 1	Option 2	Option 3
1st 3 Ingredients	Chicken meal, rice, oats	Chicken, rice, oats	Prefer not to choose
Protein Percentage	35%	20%	—
Price	$110	$80	—
I would choose	○ Chicken meal (1)	○ Chicken (2)	○ Prefer not to choose (3)

Page Break

Choice Task 13: Please select the product you would most like to purchase

$USD per 30 lb bag of commercial dry dog food

Attribute / Choice	Option 1	Option 2	Option 3
1st 3 Ingredients	Peas, rice, oats	Chicken meal, rice, oats	Prefer not to choose
Protein Percentage	35%	20%	—
Price	$95	$110	—
I would choose	○ Peas (1)	○ Chicken meal (2)	○ Prefer not to choose (3)

Page Break

Choice Task 14: Please select the product you would most like to purchase

$USD per 30 lb bag of commercial dry dog food

**Attribute / Choice**	**Option 1**	**Option 2**	**Option 3**
1st 3 Ingredients	Peas, rice, oats	Chicken, rice, oats	Prefer not to choose
Protein Percentage	35%	20%	—
Price	$95	$110	—
I would choose	○ Peas (1)	○ Chicken (2)	○ Prefer not to choose (3)

Page Break

Choice Task 15: Please select the product you would most like to purchase
$USD per 30 lb bag of commercial dry dog food

Attribute / Choice	Option 1	Option 2	Option 3
1st 3 Ingredients	Chicken, rice, oats	Peas, rice, oats	Prefer not to choose
Protein Percentage	35%	20%	—
Price	$80	$95	—
I would choose	○ Chicken (1)	○ Peas (2)	○ Prefer not to choose (3)

Page Break

Choice Task 16: Please select the product you would most like to purchase

$USD per 30 lb bag of commercial dry dog food

**Attribute / Choice**	**Option 1**	**Option 2**	**Option 3**
1st 3 Ingredients	Peas, rice, oats	Chicken, rice, oats	Prefer not to choose
Protein Percentage	35%	20%	—
Price	$95	$80	—
I would choose	○ Peas (1)	○ Chicken (2)	○ Prefer not to choose (3)

Page Break

Choice Task 17: Please select the product you would most like to purchase

$USD per 30 lb bag of commercial dry dog food

**Attribute / Choice**	**Option 1**	**Option 2**	**Option 3**
1st 3 Ingredients	Chicken meal, rice, oats	Peas, rice, oats	Prefer not to choose
Protein Percentage	35%	20%	—
Price	$80	$110	—
I would choose	○ Chicken meal (1)	○ Peas (2)	○ Prefer not to choose (3)

Page Break

Choice Task 18: Please select the product you would most like to purchase
$USD per 30 lb bag of commercial dry dog food

Attribute / Choice	Option 1	Option 2	Option 3
1st 3 Ingredients	Chicken, rice, oats	Chicken meal, rice, oats	Prefer not to choose
Protein Percentage	35%	20%	—
Price	$110	$80	—
I would choose	○ Chicken (1)	○ Chicken meal (2)	○ Prefer not to choose (3)

End of Block: Choice Tasks

Start of Block: Main Questions Cont.

Q30 Select the reasons why you think protein is important in a dog's diet. Select all that apply

- ☐ I do not think protein is important in a dog's diet
- ☐ Protein is important for lean muscle
- ☐ Protein is important to support a dog's activity
- ☐ Protein is important to support immune function
- ☐ Protein supports healthy skin and coat
- ☐ Protein satisfies a dog's hunger

Page Break

Data quality is very important to us. To show that you are paying attention, please select "organic".

- Omnivorous
- Whole grain
- Organic
- Vegetarian
- Gluten-free

Page Break

Q23 Which of the following dog diets has supplemented amino acids added to it?

- ○ Chicken, rice, lentils, blueberries, fish oil, L-lysine, D-calcium, DL-methionine
- ○ Chicken, rice, lentils, blueberries, fish oil, thiamine mononitrate, biotin, D-calcium
- ○ Chicken, rice, lentils, blueberries, fish oil, glucosamine, choline chloride, biotin
- ○ Chicken, rice, lentils, blueberries, fish oil, curcumin, thiamine mononitrate, niacin
- ○ I'm not sure what an amino acid is

Page Break

Q24 Rate the nutritional quality of the following protein sources for a dog.

	Terrible (1)	Poor (2)	Average (3)	Good (4)	Excellent (5)
Raw chicken	○	○	○	○	○
Cooked chicken	○	○	○	○	○
Beef	○	○	○	○	○
Pork	○	○	○	○	○
Peas	○	○	○	○	○
Chicken meal	○	○	○	○	○
Chicken by-products	○	○	○	○	○
Hydrolyzed protein	○	○	○	○	○
Insect	○	○	○	○	○
Beans (i.e. chickpeas, black beans, kidney beans, pinto beans, etc)	○	○	○	○	○

Page Break

Q25 On a scale, rate how true you believe the following statements are.

	Definitely false (1)	Probably false (2)	Neither true nor false (3)	Probably true (4)	Definitely true (5)
Diet can play a role in increasing or decreasing the risk of disease.	o	o	o	o	o
The amount of protein in the diet is more important than the source of protein, given it meets nutrient requirements.	o	o	o	o	o
The amino acid digestibility of protein sources is relevant to their nutritional quality	o	o	o	o	o

Page Break

Q26 How would you describe your dog's activity level?

- ◯ Couch potato
- ◯ Minimally active
- ◯ Moderately active (daily intentional activity such as walks)
- ◯ Very active
- ◯ My dog is an athlete
- ◯ I'm not sure

Page Break

Q27 How much physical activity (including walks) does your dog get in a typical day?

- None
- 0-15 minutes
- 15-30 minutes
- 30-60 minutes
- 60-90 minutes
- More than 90 minutes

Page Break

Q28 Do you believe your dog is an ideal body weight?

- Yes
- No
- Not sure

Page Break

Q29 Has anyone, including your veterinarian, ever told you that your dog is overweight?

- ○ Yes
- ○ No

End of Block: Main Questions Cont.

Start of Block: Main Questions Cont.- Consumers

Q30 Please rate the following statements based on how relatable it is to you.

	Does not describe me (1)	Describes me slightly well (2)	Describes me moderately well (3)	Describes me very well (4)	Describes me extremely well (5)
I restrict my dog’s food intake to manage its weight	o	o	o	o	o
I follow the feeding guidelines on the label	o	o	o	o	o
I feed my dog an amount of food based on my veterinarian's recommendation	o	o	o	o	o
I feed my dog an amount of food based on my breeder’s recommendation	o	o	o	o	o
I feel like I can decipher between good and poor sources of protein for my dog	o	o	o	o	o
I check the protein amount in my dog’s diet	o	o	o	o	o
I think that too much protein in my dog’s diet can contribute to climate change	o	o	o	o	o

Page Break

Q31 Would you consider feeding your dog a diet that includes alternative protein sources? If so, please select all from the list below that you would consider.

- ☐ Hydrolyzed protein
- ☐ Legumes ie: soy, chickpeas, peas, lentils etc.
- ☐ Yeast proteins
- ☐ Insect meal
- ☐ Algae
- ☐ Cultured meat
- ☐ Seaweed
- ☐ Other
- ☐ I would not feed my dog alternative protein sources, they need animal meat only

Page Break

Q32 Have you ever looked for different protein sources in your dog's food because they have experienced any of the following conditions? Select all that apply.

- ☐ Itchy skin
- ☐ Hair loss
- ☐ Smelly skin
- ☐ Soft stool
- ☐ Smelly stool
- ☐ None of the above
- ☐ Other

Page Break

Q33 How much impact do the following options have on your decision when buying dog food? (From 0 to 5, with 0 being no impact, 3 being may consider and 5 meaning it is a priority deciding factor)

	0 (1)	1 (2)	2 (3)	3 (4)	4 (5)	5 (6)
Raw-meat based	○	○	○	○	○	○
Contains fresh meat	○	○	○	○	○	○
Animal meat 1st ingredient	○	○	○	○	○	○
Protein #1 ingredient	○	○	○	○	○	○
No animal by-product	○	○	○	○	○	○
High protein diet	○	○	○	○	○	○
High-quality protein	○	○	○	○	○	○
Excellent protein sources	○	○	○	○	○	○
No pulse ingredients	○	○	○	○	○	○
Humanely raised animals	○	○	○	○	○	○
Sustainably grown crops	○	○	○	○	○	○
100% traceable ingredients	○	○	○	○	○	○
Responsibly sourced ingredients (ie: cage-free eggs)	○	○	○	○	○	○

Page Break

Q34 Where do you get information about dog food from? Select all that apply

- ▢ Veterinarian
- ▢ Friends and family
- ▢ Breeder
- ▢ Animal shelter/rescue
- ▢ Pet store
- ▢ Online research
- ▢ Online from Company websites
- ▢ TV
- ▢ Other

Page Break

Q35 Where do you primarily buy your dog's food from?

- Pet store
- Grocery store
- Vet clinic
- Online
- Other

Page Break

Q36 What was your motivation for getting a dog? Select all that apply

- ▢ Security
- ▢ Companionship
- ▢ Sporting use
- ▢ Working use (ie: herding, sledding)
- ▢ Emotional/mental support
- ▢ For your children
- ▢ Company for another pet
- ▢ Leisure/fun
- ▢ Other

End of Block: Main Questions Cont.- Consumers

Start of Block: Demographics

QD1 What state do you live in?

- ○ Alabama
- ○ Alaska
- ○ Arizona
- ○ Arkansas
- ○ California
- ○ Colorado
- ○ Connecticut
- ○ Delaware
- ○ Florida
- ○ Georgia
- ○ Hawaii
- ○ Idaho
- ○ Illinois
- ○ Indiana
- ○ Iowa
- ○ Kansas
- ○ Kentucky
- ○ Louisiana
- ○ Maine

- Maryland
- Massachusetts
- Michigan
- Minnesota
- Mississippi
- Missouri
- Montana
- Nebraska
- Nevada
- New Hampshire
- New Jersey
- New Mexico
- New York
- North Carolina
- North Dakota
- Ohio
- Oklahoma
- Oregon
- Pennsylvania
- Rhode Island

- South Carolina
- South Dakota
- Tennessee
- Texas
- Utah
- Vermont
- Virginia
- Washington
- West Virginia
- Wisconsin
- Wyoming

Page Break

QD2 What is your highest level of education?

- ○ Less than a high school diploma
- ○ High school degree or equivalent (e.g. GED)
- ○ College degree
- ○ Bachelor's degree (e.g. BA, BSc)
- ○ Master's degree (e.g. MA, MSc, MEd)
- ○ Professional degree (e.g. MD, DDS, DVM)
- ○ Doctorate (e.g. PhD, EdD)

Page Break

QD3 What is the total combined income of your household after taxes?

- $0 - $24,999
- $25,000 - $49,999
- $50,000 - $74,999
- $75,000 - $99,999
- $100,000 - $124,999
- $125,000 - $149,999
- $150,000 or more

End of Block: Demographics
